# Interferon-Gamma and Nitric Oxide Synthase 2 Mediate the Aggregation of Resident Adherent Peritoneal Exudate Cells: Implications for the Host Response to Pathogens

**DOI:** 10.1371/journal.pone.0128301

**Published:** 2015-06-01

**Authors:** Bhagawat S. Chandrasekar, Shikha Yadav, Emmanuel S. Victor, Shamik Majumdar, Mukta Deobagkar-Lele, Nitin Wadhwa, Santosh Podder, Mrinmoy Das, Dipankar Nandi

**Affiliations:** 1 Department of Biochemistry, Indian Institute of Science, Bangalore, India; 2 Centre for Infectious Disease Research (CIDR), Indian Institute of Science, Bangalore, India; National University of Ireland Galway, IRELAND

## Abstract

Interferon-gamma (Ifnγ), a key macrophage activating cytokine, plays pleiotropic roles in host immunity. In this study, the ability of Ifnγ to induce the aggregation of resident mouse adherent peritoneal exudate cells (APECs), consisting primarily of macrophages, was investigated. Cell-cell interactions involve adhesion molecules and, upon addition of Ifnγ, CD11b re-localizes preferentially to the sites of interaction on APECs. A functional role of CD11b in enhancing aggregation is demonstrated using Reopro, a blocking reagent, and siRNA to *Cd11b*. Studies with NG-methyl-L-arginine (LNMA), an inhibitor of Nitric oxide synthase (Nos), NO donors, e.g., S-nitroso-N-acetyl-DL-penicillamine (SNAP) or Diethylenetriamine/nitric oxide adduct (DETA/NO), and *Nos2* ^-/-^ mice identified Nitric oxide (NO) induced by Ifnγ as a key regulator of aggregation of APECs. Further studies with *Nos2* ^-/-^ APECs revealed that some Ifnγ responses are independent of NO: induction of MHC class II and CD80. On the other hand, Nos2 derived NO is important for other functions: motility, phagocytosis, morphology and aggregation. Studies with cytoskeleton depolymerizing agents revealed that Ifnγ and NO mediate the cortical stabilization of Actin and Tubulin which contribute to aggregation of APECs. The biological relevance of aggregation of APECs was delineated using infection experiments with *Salmonella* Typhimurium (*S*. Typhimurium). APECs from orally infected, but not uninfected, mice produce high amounts of NO and aggregate upon *ex vivo* culture in a Nos2-dependent manner. Importantly, aggregated APECs induced by Ifnγ contain fewer intracellular *S*. Typhimurium compared to their single counterparts post infection. Further experiments with LNMA or Reopro revealed that both NO and CD11b are important for aggregation; in addition, NO is bactericidal. Overall, this study elucidates novel roles for Ifnγ and Nos2 in regulating Actin, Tubulin, CD11b, motility and morphology during the aggregation response of APECs. The implications of aggregation or “group behavior” of APECs are discussed in the context of host resistance to infectious organisms.

## Introduction

Cell-cell interactions, an important mode of communication, are fundamental to an organism’s ability to survive and grow. In the context of immune responses, physical interactions and biochemical communications between different leukocytes are essential. Interactions involving immune cells, e.g. APCs with CD4^+^ T cells, cytotoxic T cells with target cells etc. are characterized by the formation of ‘immunological synapses.’ These integrate signals and promote molecular interactions for an effective immune response [1]. Immune cells of both innate and adaptive arms are known to interact with endothelial cells in the vasculature before extravasating to the sites of inflammation or infection [2]. Thus, cell-cell interactions involving immune cells are essential for the development of host immunity.

Macrophages are present in almost every tissue and are important in the maintenance of tissue homeostasis, mediation of inflammatory responses, wound healing etc [3,4]. Tissue-resident macrophages are extremely heterogeneous, act in accordance to the ‘micro-anatomical niche’ of residence and exhibit a wide variety of functions [5]. Macrophages are known to interact with multiple cell types in different tissues to mediate host responses. The peritoneal cavity of mice contains numerous types of immune cells and is commonly used as a source for macrophages. In fact, much of our knowledge of macrophage biology is derived from studies on cells from this source [6]. In the bone marrow, macrophages interact with erythrocytes and phagocytose extruded nuclear material from developing erythrocytes [7]. Kupffer cells, the resident liver macrophages, interact with hepatocytes and platelets to regulate their functions and initiate responses to blood borne pathogens [8,9]. Macrophages in the bone, known as osteoclasts, constantly interact with osteoblasts to shape bone homeostasis and contribute to the maintenance of the ‘hematopoietic niche’ in the marrow, an aspect highlighted during ‘osteopetrosis’ [10]. Therefore, interactions of macrophages with other cell types determine their physiological roles during homeostasis and inflammation. However, attempts to understand macrophage-macrophage interactions, the underlying mechanisms and functional contributions have been remote.

Among the activators of macrophages, Interferon-gamma (Ifnγ), earlier identified as ‘Macrophage Activating Factor’ [11], is one of the most potent inducers of antimicrobial responses. Ifnγ activates the Janus Activating Kinase—Signal Transducer and Activator of Transcription pathway and modulates a wide variety of host responses [12]. Indeed, humans or mice lacking Ifnγ or its receptor are highly susceptible to infections by less virulent intracellular pathogens [13,14]. Ifnγ can also affect cell-cell interactions by modulating the expression of cell surface adhesion molecules [15,16]. In this study, we show that Ifnγ induces cellular interactions and formation of stable aggregates of primary mouse resident adherent peritoneal exudate cells (APECs). Furthermore, the functional contributions of Nitric oxide synthase 2 (Nos2), cytoskeletal proteins and the cell surface integrin CD11b to this phenomenon are delineated.

To investigate the physiological relevance of our observations, we utilized a mouse model of infection and inflammation using *Salmonella* Typhimurium (*S*. Typhimurium). This intracellular Gram negative bacterium is closely related to *S*. Typhi, the causative agent for typhoid fever in humans [17]. Also, *Salmonella* infection is a major public health problem especially in immuno-compromised individuals, such as HIV infected cohorts, in Africa [18]. Hence, understanding the various immune parameters that may influence the outcome of *Salmonella* infection is important. In this study, implications of the aggregation phenotype of APECs during infection with *S*. Typhimurium were investigated. Overall, our studies provide novel mechanistic insights into the roles of Nos2 in mediating morphology, motility and aggregation behavior of APECs upon Ifnγ stimulation and in response to infection with *S*. Typhimurium.

## Materials and Methods

### Chemicals and other reagents

Recombinant mouse Ifnγ was purchased from PeproTech Asia, Israel. Lipopolysaccharide (LPS) from *Escherichia coli* serotype O55:B5, NG-methyl-L-arginine (LNMA), S-nitroso-N-acetyl-DL-penicillamine (SNAP), Diethylenetriamine/nitric oxide adduct (DETA/NO), Polyethyleneglycol-Catalase (PC), Cytochalasin D (Cyt D) and Colcemid (Col) were from Sigma Aldrich, USA. Reopro was obtained from Eli Lilly and Company, India and scrambled and *Cd11b* specific siRNA were procured from Dharmacon, USA.

### Bacterial strain and culture

A single colony of *S*. Typhimurium NCTC 12023 was cultured overnight in 3 ml Luria broth and, subsequently, inoculated into 50 ml sterile Luria broth at 0.2% and grown for 3–4 h (log phase culture for oral infection of mice) or overnight (stationary phase culture for infecting APECs) at 37°C, 160 rpm [19]. Bacterial cells were centrifuged, washed and re-suspended in sterile PBS for further experiments. For specific experiments, *S*. Typhimurium expressing green fluorescent protein (Sal-GFP) was generated by transforming *S*. Typhimurium with the plasmid pProEx-HT (Invitrogen, USA) containing GFP (a kind gift from D. Saini, IISc) and cultured as mentioned above for *in vitro* infections of APECs.

### Animal ethics statement

All mice experiments were conducted in compliance with the Ministry of Environment and Forests Act (Government of India) regarding the breeding of and experiments on animals (control and supervision) rules, 1998 and in accordance with the guidelines stated by the Institutional Animal Ethics Committee, IISc. Mice were bred and maintained at the Central Animal Facility of IISc (Registration number: 48/1999/CPCSEA, dated 1/3/1999), accredited to the Ministry of Environment and Forests, Government of India. The experimental protocols were approved by the ‘Committee for Purpose and Control and Supervision of Experiments on Animals’ (CPCSEA) and the approved permit number was CAF/Ethics/155/2009. The details of these national guidelines can be seen in the following web site: http://envfor.nic.in/division/committee-purpose-control-and-supervision-experiments-animals-cpcsea.

### Mice and infections

All the experiments were performed using ~6–8 week old C57BL/6 and C57BL/6. *Nos2* ^-/-^ (*Nos2* ^-/-^) mice obtained from the Central Animal Facility of the Indian Institute of Science, Bangalore. Mice were orally fed ~10^8^ colony forming units (CFU) of *S*. Typhimurium in a final volume of 0.5 ml PBS. On indicated days post infection, mice were sacrificed by euthanization with CO_2_. Subsequently, the peritoneal lavage containing cells was collected using RPMI containing 5% FCS supplemented with antibiotics. Each group of mice consisted of 4–10 mice and care was taken to keep the cells isolated from individual mice as separate and not pooled. APECs were used for analysis of cell surface markers and the sera were used for estimating cytokine amounts by ELISA [19].

### Cell culture

APECs were isolated as previously described [19] using RPMI 1640 (Sigma) supplemented with 5% heat inactivated FCS (Invitrogen, USA), 5 μM β-mercaptoethanol (Sigma), 100 μg/ml penicillin, 250 μg/ml streptomycin, 50 μg/ml gentamycin (Himedia Laboratories, India) and plated at a cell density of ~ 2 x 10^5^ or ~ 1 x 10^6^ cells per well in a 96 well plate or 24 well plate, respectively. The cells were allowed to adhere for 45 min at 37°C in a humidified CO_2_ incubator (Sanyo, UK) and the non-adherent cells were washed away using phosphate buffered saline, PBS (Sigma, USA). APECs were pretreated with Cyt D or Col for 6 h before the addition of Ifnγ as indicated. SNAP or DETA/NO were used at concentrations that resulted in amounts of nitrite similar to that observed in Ifnγ treated C57BL/6 APECs. The number of live cells was determined by trypan blue (0.4% solution w/v) exclusion assay using a hemocytometer.

### Bright field and fluorescence imaging

Bright field images were acquired using 1X71 inverted microscope (Olympus, Japan), DMI6000B inverted live imaging or TCS SP5 upright confocal microscope (Leica, Germany) at magnifications as indicated. The surface and intracellular fluorescence imaging was performed on APECs stained on glass cover-slips after appropriate treatments. Imaging was performed using the ApoTome.2 inverted fluorescence microscope at 63X or LSM 510 META inverted confocal microscope (Zeiss, Germany) at 100X oil immersion objectives. The images were recorded using the Axio Vision, Kombi-FCS LSM–META or LAS AF softwares. Multiple fields across each sample were acquired.

### Estimation of intracellular ROS

Live cells were treated with 5 μM of 2′,7′-Dichlorodihydrofluorescein Diacetate (DCFDA) (Merck-Millipore, USA) and bisBenzimide H 33342 (Sigma) at 100 nM dilution in PBS for 15 min at 37°C. The amount of total fluorescence was obtained using Infinite 200 fluorescence microplate reader (Tecan, Switzerland) and the analysis was performed as previously reported (19). The extent of DCFDA fluorescence in each well was normalized to the corresponding extent of nuclear staining with bisBenzimide H 33342. The fluorescence of DCFDA in untreated cells was considered as control and fluorescence from other treatments or time points post treatment were calculated as fold change with respect to control.

### Nitrite estimation

The amounts of nitrite in the supernatant (50 μl) of APECs was estimated using Griess reagent (100 μl) that constituted 1% Sulfanilamide and 0.1% N-(1-Naphthyl)ethylenediamine (Sigma) in 2.5% orthophosphoric acid (Merck—Millipore) containing water as described previously (19). Also, a standard with sodium nitrite (0.2–208 μM) was used to quantify the amounts of nitrite in the supernatant. Optical density readings at 550 nm were obtained using VersaMax ELISA plate reader (Molecular Devices, USA).

### Environmental Scanning Electron Microscopy

APECs, post various treatments, were fixed using 4% Paraformaldehyde (PFA) and 1% Glutaraldehyde (Sigma) for 10 min at 37°C. The APECs were extensively washed with PBS and, subsequently, using doubly autoclaved distilled water. The cover-slips containing the samples were air dried and loaded onto stubs using adhesive tape. The images are acquired at high vacuum mode at 25 kV and at a magnification of 5000X using FEI Environmental Scanning Electron Microscopy (ESEM) Quanta 200 microscope (FEI, USA).

### Intracellular fluorescence staining for proteins

After appropriate treatments, APECs were fixed using 4% PFA and 0.5% Glutaraldehyde in PBS for 10 min at 37°C and washed well with PBS. Cells were permeabilized using ice cold Acetone for 15 min. The samples were blocked with blocking buffer containing 5% FCS in PBS. Antibodies specific to α-Tubulin (Santa Cruz Biotechnology, USA), Lamp1 (clone 1D4B developed by J. Thomas August was obtained from the Developmental Studies Hybridoma Bank under the auspices of the NICHD and maintained by the University of Iowa, IA 52242), Alexa Fluor 488 Phalloidin (Life Technologies, USA), Nos2 (Millipore, USA) and Arginase1 (Millipore) were used at 1:250–1:500 dilution in blocking buffer and added to permeabilized cells for 30 min at 37°C. After thorough washing with PBS, respective secondary antibodies conjugated with Phycoerythrin (Jackson Laboratories, USA) were used at 1:400 dilution in blocking buffer and added to samples for 30 min at 37°C. Cells were washed well and stained for nucleus using bisBenzimide H 33342 at 500 nM dilution for 15 min at 37°C. The images were captured using a Zeiss 510 Meta confocal microscope.

### Fluorescence cell surface staining

APECs were first blocked with blocking buffer containing 5% FCS along with 2.5% BSA in the presence of functional anti-CD16 (eBioscience, USA) at 1:200 dilution for 15 min at 4°C. Direct conjugated antibodies to Major Histocompatibility Complex encoded class I (MHC class I) (FITC), class II (FITC), CD11b (PE), F4/80 (PE), Gr1 (FITC), CD3 (PE), B220 (FITC), CD80 (FITC), CD86 (FITC), CD45 (FITC) and P-Selectin (PE) (eBioscience, USA) were diluted in blocking buffer at 1:200 dilution and added to APECs for 30 min at 4°C. Wherever indicated, 250 μl of cellular supernatant containing monoclonal antibodies, anti-lymphocyte function associated antigen 1 (Lfa1) (M17-4-4 clone) and anti- Intercellular adhesion molecule 1 (Icam1) (YN1/1.7.4 clone), were used along with 100 μl of blocking buffer for 30 min at 4°C. Anti-E-Selectin (P2H3 clone developed by Elizabeth A. Wayner/Gregory Vercellotti was obtained from the Developmental Studies Hybridoma Bank developed under the auspices of the NICHD and maintained by The University of Iowa, Department of Biology, Iowa City, IA 52242) was used at 1:200 dilution in the blocking buffer for 30 min at 4°C. After thorough washing of cells post treatment, specific secondary antibodies tagged with FITC or PE to Lfa1 and Icam1 wherever necessary were diluted 1:200 in the blocking buffer and added to cells for 30 min at 4°C. The cells were washed thoroughly and stained for nucleus using bisBenzimide H 33342 at 500 nM dilution for 15 min at 4°C and fixed using 4% PFA for 10 min at 37°C for microscopy only. Images were acquired using Zeiss 510 Meta confocal microscope. Wherever necessary, the amount of total fluorescence was obtained using Infinite 200 pro fluorescence microplate reader and represented as fold change with respect to untreated C57BL/6 control APECs. For fluorescence measurement and flow cytometry, cells were finally fixed using 4% PFA for 10 min at 37°C and washed well with PBS before acquiring the same using BD FACS Verse (BD Biosciences, USA) and analyzed using WinMdi 2.8 software.

### Live cell imaging and motility

Imaging of APECs was performed at 40X using Leica DMI6000B live imaging microscope (Leica, Germany) at 37°C along with 5% CO_2_. Images were acquired every 5 min and made into a video using the LAS AF software. The videos were analyzed using the manual tracking method plugged into the Image J software. Briefly, the motility of a minimum of 25 APECs were tracked across 6 h, i.e. 18–24 h post treatment unless otherwise mentioned and represented as a single video. The changes in the X and Y coordinates in each image of the video for each cell were used to calculate their independent velocities. The average velocities exhibited by untreated C57BL/6 APECs were considered as 100% and the other conditions were represented in comparison to the same as percentages.

### Intracellular infection of APECs with Sal–GFP

For the *in vitro* infection assay, 0.2 x 10^6^ APECs were plated per well in 96 well plates in RPMI with 10% FCS without antibiotics. APECs were infected with an overnight culture of Sal-GFP at a multiplicity of infection of 1:50 for 45 min, unless otherwise mentioned. Cells were washed with PBS post infection and 100 μg/ml gentamycin was added into the medium for 45 min. Cells were washed again with PBS and cultured in RPMI with 10% FCS containing 25 μg/ml gentamycin. At 2 h post-infection, the CFU burden was elucidated and interpreted as phagocytic uptake by APECs. APECs were treated with or without 25 U/ml of Ifnγ for 24 h as well as in the presence or absence of LNMA (200 μM) post infection. The number of Sal-GFP per cell was elucidated by staining cells for Lamp1 (red, cytosolic) and bisBenzamide H 33342 (blue, nuclear) and counting the total number of Sal-GFP in each field and divided by the total number of cells in the field manually across different Z positions using a 63X magnification using the ApoTome.2 fluorescence microscope. For finer details, images were acquired at 100X magnification using a Zeiss confocal microscope.

### ELISA

The amounts of cytokines, Ifnγ (Cat. No. 13-7312-68C), Il6 (Cat. No. 33-7062-68), Tnfα (Cat. No. 13-7341-68), in the sera (1:5 dilution) were estimated using ELISA kits from eBioscience, USA. Protocols recommended by the manufacturers were followed to quantify the amounts of cytokines in each sample. TMB was used as the substrate for the development and optical density readings were obtained at 450 nm using VersaMax ELISA plate reader. For each of the cytokines, appropriate standards (~31 to 1000 pg/ml) and blanks were used.

### Phagocytosis assay

Fluorescent yellow green latex beads of size 2 μm (Sigma) were added at a concentration of 10 μg/ml to APECs and incubated for 30 min at 37°C. Further the cells were washed with PBS (five times) to remove any excess beads that remained extracellular, before staining with bisBenzamide. The amount of total fluorescence was obtained using Infinite 200 pro fluorescence microplate reader and represented fold change with respect to the untreated C57BL/6 APECs.

### Statistical analysis

All the data were plotted using GraphPad Prism 5 software and the data is presented as the mean ± standard error (SE). The significance was obtained by performing one way analysis of variance (ANOVA) among the test groups and *p*-values less than 0.05, 0.01 and 0.001 have been represented as *, ** and *** respectively in comparison to untreated control. In case other controls were used for comparison, the usage of additional symbols for the same are mentioned in the figure legends.

## Results

### Ifnγ induces aggregation of APECs in a CD11b-dependent manner

**Fig 1:**
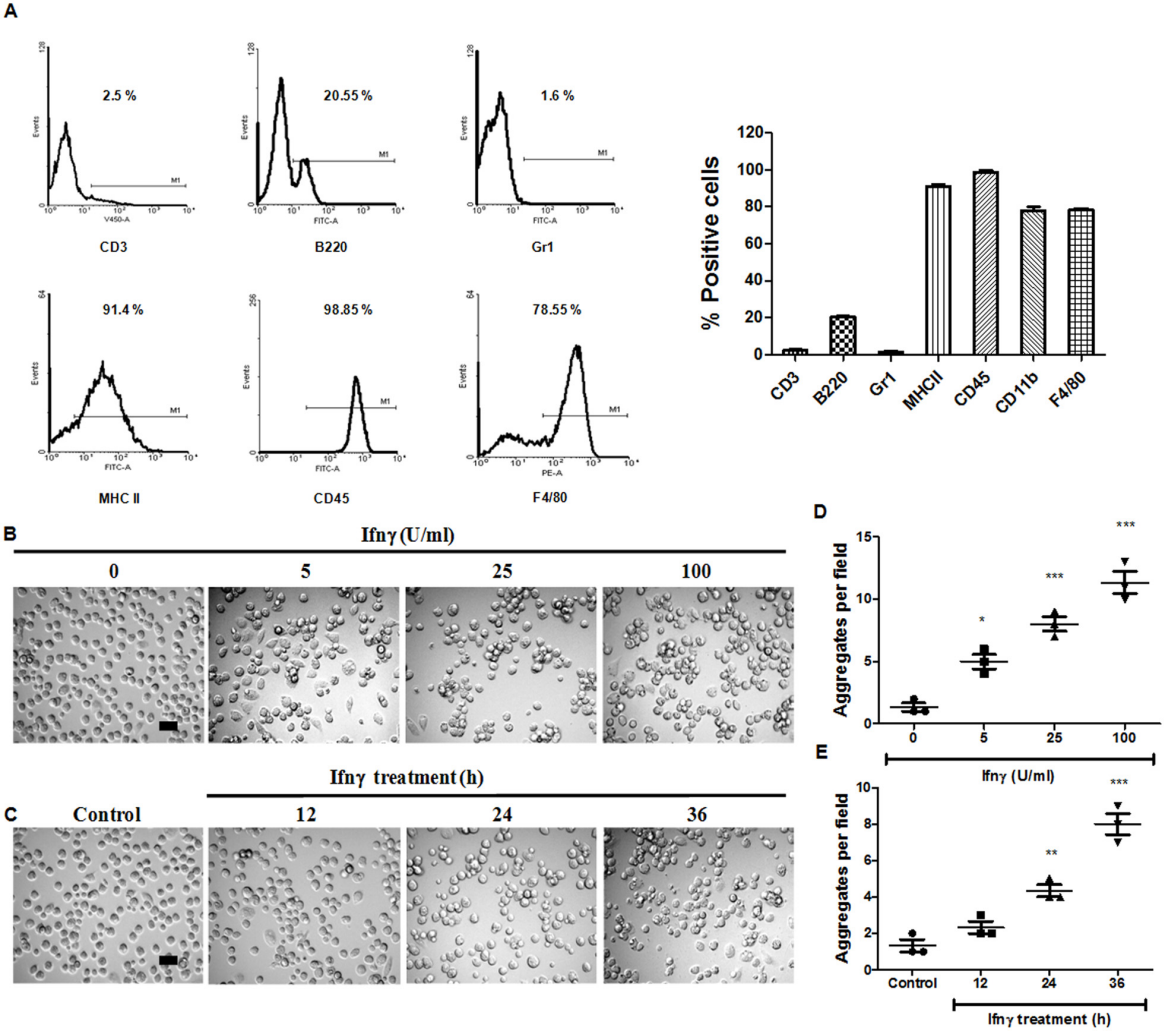
Ifnγ induces APECs to aggregate. APECs were isolated from C57BL/6 mice, cultured in tissue culture media for ~ 24 h and were characterized by staining with a panel of antibodies to different cell surface proteins followed by FACS analysis. Representative plots and data (A) for different markers is shown which are representative of multiple experiments with APECs from 4 mice. Bright field images of APECs treated with different doses of Ifnγ for 36 h (B) and kinetics of APECs treated with 25 U/ml Ifnγ for indicated time points (C). The scale bar represents 20 μm. Quantification of the extent of APECs aggregates formed as a function of dose of Ifnγ (D) and incubation time (E). An aggregate consists of six or more interacting cells in any given field acquired at 20X magnification. The data is represented as mean ± S.E from three independent experiments.

**Fig 2:**
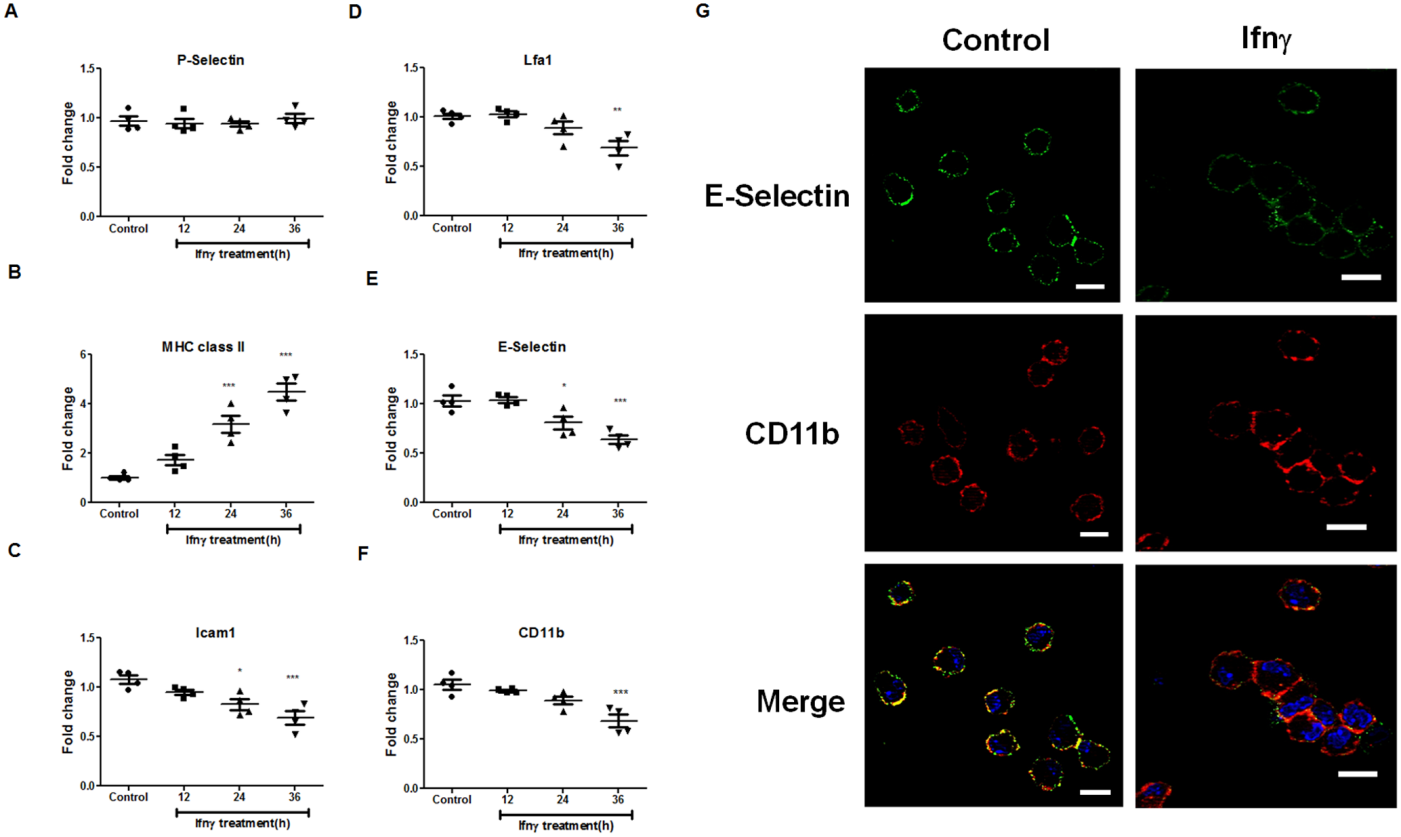
Modulation of expression of different cell surface proteins on APECs in response to Ifnγ. Kinetic analysis of cell surface amounts of P-Selectin (A), MHC class II (B), Icam1 (C), Lfa1 (D), E-Selectin (E) and CD11b (F) upon Ifnγ treatment (25 U/ml) of APECs. Fluorescence microscopic images acquired at 63X with a scale of 10 μm of E-Selectin and CD11b on the cell surface of APECs treated with 25 U/ml of Ifnγ for 36 h (G). The data is represented as mean ± S.E from two independent experiments.

We have been interested in elucidating novel functional responses to Ifnγ by tumor cells and primary APECs [19–21]. As observed in Fig 1A, there are very few T cells or neutrophils in APECs which predominantly consists of ~80% macrophages as indicated by the markers such as MHC class II, CD11b and F4/80. However, a minor population of B220 positive cells was also present in our culture system. We observed that addition of Ifnγ in a dose and time dependent manner increased the formation of aggregates of APECs. These are defined as clusters of six or more cells in close association with each other in a given field at a given time point (Fig 1B–1E). As cell-cell interactions often involve adhesion molecules, we investigated the possible regulation and roles of cell surface selectins and integrins. Ifnγ is known to induce the expression of MHC molecules [12] and modulate the expression of adhesion molecules [15,16]. Therefore, the quantitative expression of different cell surface molecules in response to Ifnγ treatment was analyzed in a kinetic manner. The addition of Ifnγ did not affect cell surface amounts of P-Selectin, but led to an increase in cell surface expression of MHC class II molecule (Fig 2A and 2B). However, Ifnγ led to a modest decrease in the expression of Icam1, Lymphocyte function associated antigen 1 (Lfa1), E-selectin and CD11b (Fig 2C–2F). Further, fluorescence microscopic analysis of APECs in response to Ifnγ treatment revealed preferential cell surface localization of CD11b to the sites of interaction of APECs in aggregates (Fig 2G). It is important to point out that although CD11b appeared enriched at the sites of cell-cell interaction in fluorescence imaging of a single Z plane (Fig 2G), overall CD11b amounts on the cell surface of APECs were reduced upon Ifnγ treatment (Fig 2F). Overall, the expression of several adhesion molecules was marginally reduced and is accompanied by preferential localization of CD11b to the sites of interaction in aggregated APECs.

**Fig 3:**
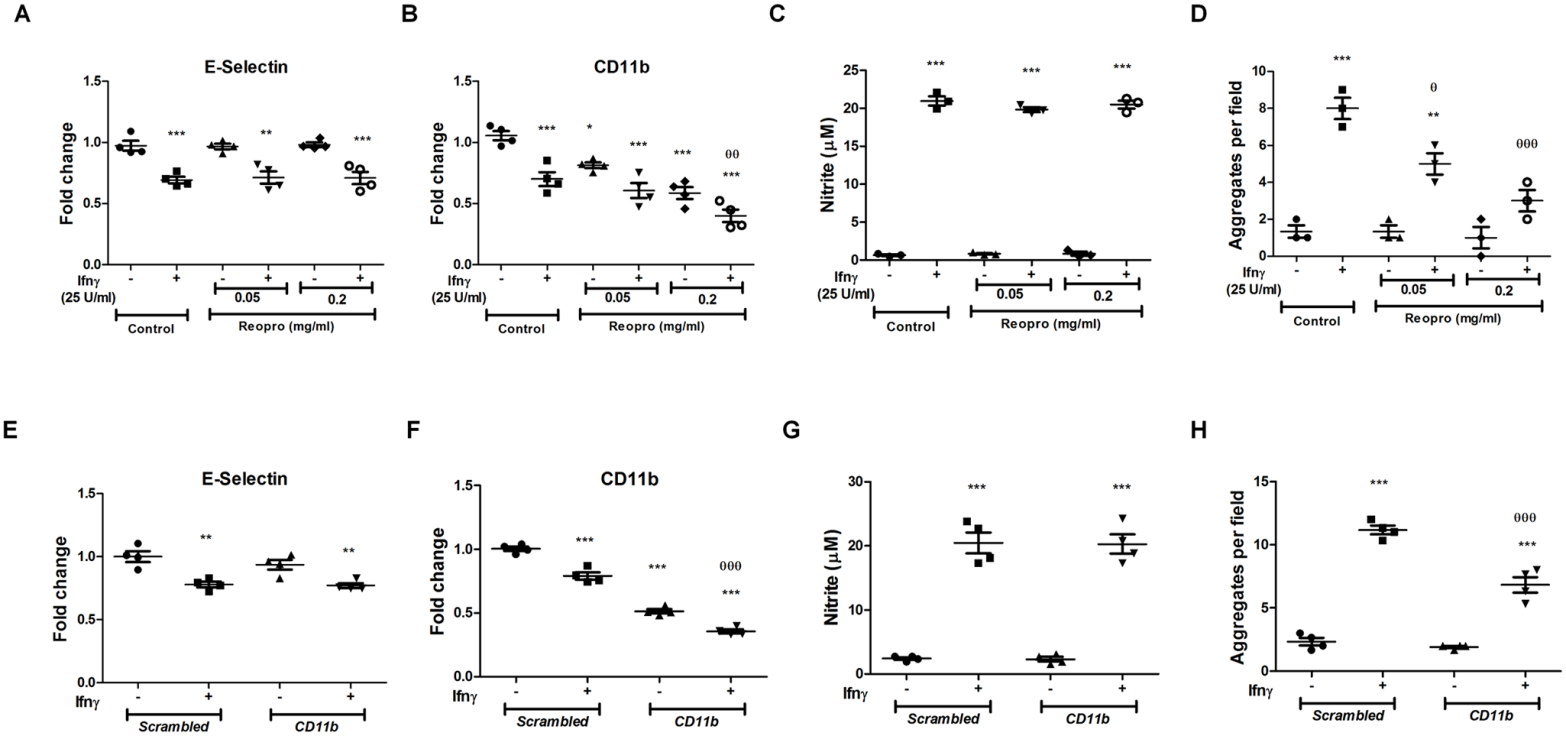
CD11b is required for aggregation of APECs in response to Ifnγ. The relative amounts of E-Selectin (A, E) and CD11b (B, F) on APECs upon treatment with Ifnγ (25 U/ml) in the absence or presence of indicated amounts of Reopro (A-D) or siRNA to *Cd11b* (E-H) was measured after 36 h. The amount of nitrite (C,G) and cell aggregates (D,H) upon 25 U/ml of Ifnγ treatment in the absence or presence of indicated doses of Reopro or 200 nM siRNA to *Cd11b* is shown. The data is represented as mean ± S.E from three independent experiments and control refers to untreated cells alone. The significance with respect to untreated controls and Ifnγ treated C57BL/6 APECs controls are represented as * and θ respectively.

To study the functional contribution of CD11b, studies with Reopro [22], a purified Fab against glycoprotein GP IIb/IIIa that also blocks CD11b, were performed. The addition of Reopro reduced CD11b, but not E-Selectin, detection on the cell surface after 36 h (Fig 3A and 3B). Importantly, Reopro treatment in a dose dependent manner reduced Ifnγ induced aggregation of APECs, but not nitrite production (Fig 3C and 3D). These data were confirmed using siRNA to *Cd11b*. As oligonucleotides are known to affect immune responses [23], initial experiments were performed to determine the role of the scrambled control in modulating functions of APECs. As seen in Fig. A in S1 File, transfections with the scrambled siRNA did not alter any major responses in APECs with respect to nitrite, CD11b expression and aggregation. However, CD11b expression, but not E-Selectin, is lowered with siRNA to *Cd11b* but not the scrambled control (Fig 3E and 3F). Although Ifnγ-induced nitrite amounts remained unaffected, the number of aggregates was significantly reduced upon knockdown of CD11b (Fig 3G and 3H). Therefore, CD11b on the cell surface aids in the formation of aggregates of APECs in response to Ifnγ.

### Nos2 derived nitric oxide (NO) promotes the aggregation response of APECs to Ifnγ

**Fig 4:**
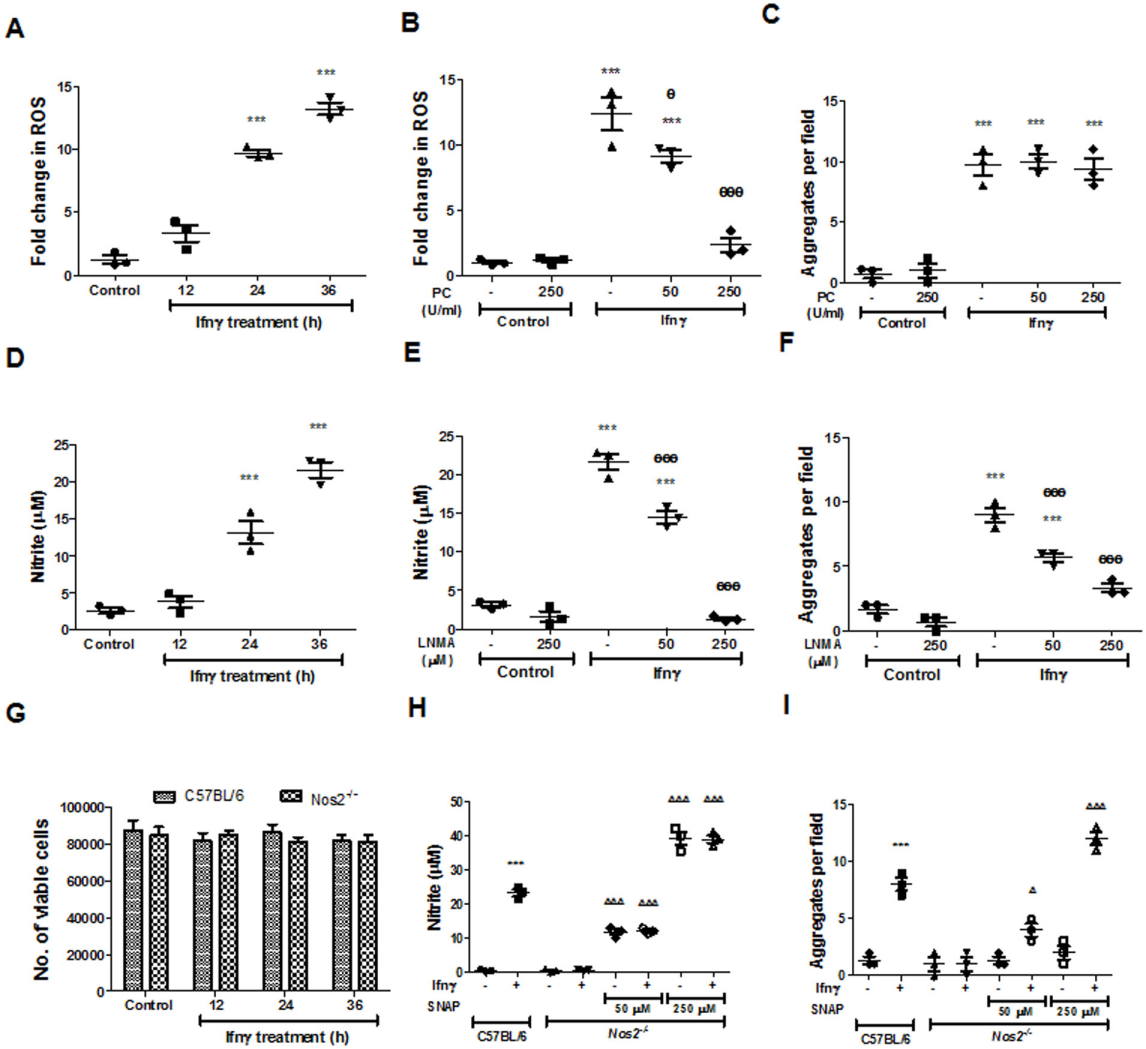
Nos2 derived NO mediates the Ifnγ induced aggregation of APECs. Kinetic analysis of amounts of ROS (A), nitrite (D) of APECs treated with 25 U/ml of Ifnγ. The amounts of ROS (B) and the number of cell aggregates (C) in APECs treated with Ifnγ in the presence or absence of indicated doses of PEG-Catalase (PC) for 36 h. The amount of nitrite (E) and the number of cell aggregates (F) of APECs treated with Ifnγ in the presence or absence of indicated doses of LNMA for 36 h. Kinetic analysis of the number of viable APECs from C57BL/6 and *Nos2* ^*-/-*^ mice left untreated or upon treatment with 25 U/ml of Ifnγ (G). The amount of nitrite (H) and the number of cell aggregates (I) from Ifnγ treated *Nos2* ^*-/-*^ APECs in the absence or presence of indicated doses of SNAP for 36 h in comparison to C57BL/6 APECs treated with 25 U/ml Ifnγ for 36 h. The data is represented as mean ± S.E from three independent experiments. The significance with respect to untreated controls, Ifnγ treated C57BL/6 APECs controls and untreated *Nos2* ^*-/-*^ APECs controls are represented as *, θ and Δ respectively.

Next, we investigated the intracellular signaling molecules that might contribute to the phenomenon of Ifnγ mediated aggregation of APECs. Ifnγ is a potent inducer of reactive oxygen species (ROS) and NO in macrophages and several Ifnγ induced responses are dependent on these molecules [19–21]. Ifnγ induced ROS in a kinetic manner, which could be quenched by exogenously added Polyethyleneglycol-Catalase (PC); however, the aggregation of APECs remained unaffected (Fig 4A–4C). On the other hand, Ifnγ treatment also induced the production of nitrite in a kinetic manner, which was inhibited by the Nos inhibitor, LNMA. Importantly, addition of LNMA inhibited Ifnγ induced aggregation of APECs in a dose dependent manner (Fig 4D–4F).

As the predominant isoform of Nos expressed in macrophages is Nos2 [24], the possible role of Nos2 generated NO in mediating Ifnγ induced APECs aggregation was investigated. First, Ifnγ treatment did not affect the viability of APECs derived from either C57BL/6 or *Nos2* ^*-/-*^ mice (Fig 4G). Second, Ifnγ induced nitrite in APECs from C57BL/6, but not from *Nos2* ^*-/-*^, mice (Fig 4H). Third and most importantly, the lack of Nos2 completely abrogated the Ifnγ induced aggregation response of APECs (Fig 4I). To study the direct contribution of NO, exogenous supplementation experiments were performed with NO donor, SNAP. It is important to point out that that the concentrations of SNAP used were ones that produced nitrite amounts similar to that seen with Ifnγ stimulation of C57BL/6 APECs (Fig 4H). Notably, *Nos2* ^*-/-*^ APECs do not form aggregates with SNAP alone; however, aggregates of *Nos2* ^*-/-*^ APECs were observed with the combination of Ifnγ and SNAP (Fig 4I). These results were confirmed using another NO donor, DETA/NO, which has a longer half life compared to SNAP [25]. As seen in Fig. B in S1 File, DETA/NO, in a dose-dependent manner, induced nitrite. However, the aggregation of *Nos2* ^-/-^ APECs was induced only with the combination of Ifnγ and higher amounts of DETA/NO. Overall, these results clearly demonstrate that the combination of Ifnγ and NO derived signals is required for aggregation of APECs.

### Nos2 regulates the amounts of cell surface CD11b

**Fig 5:**
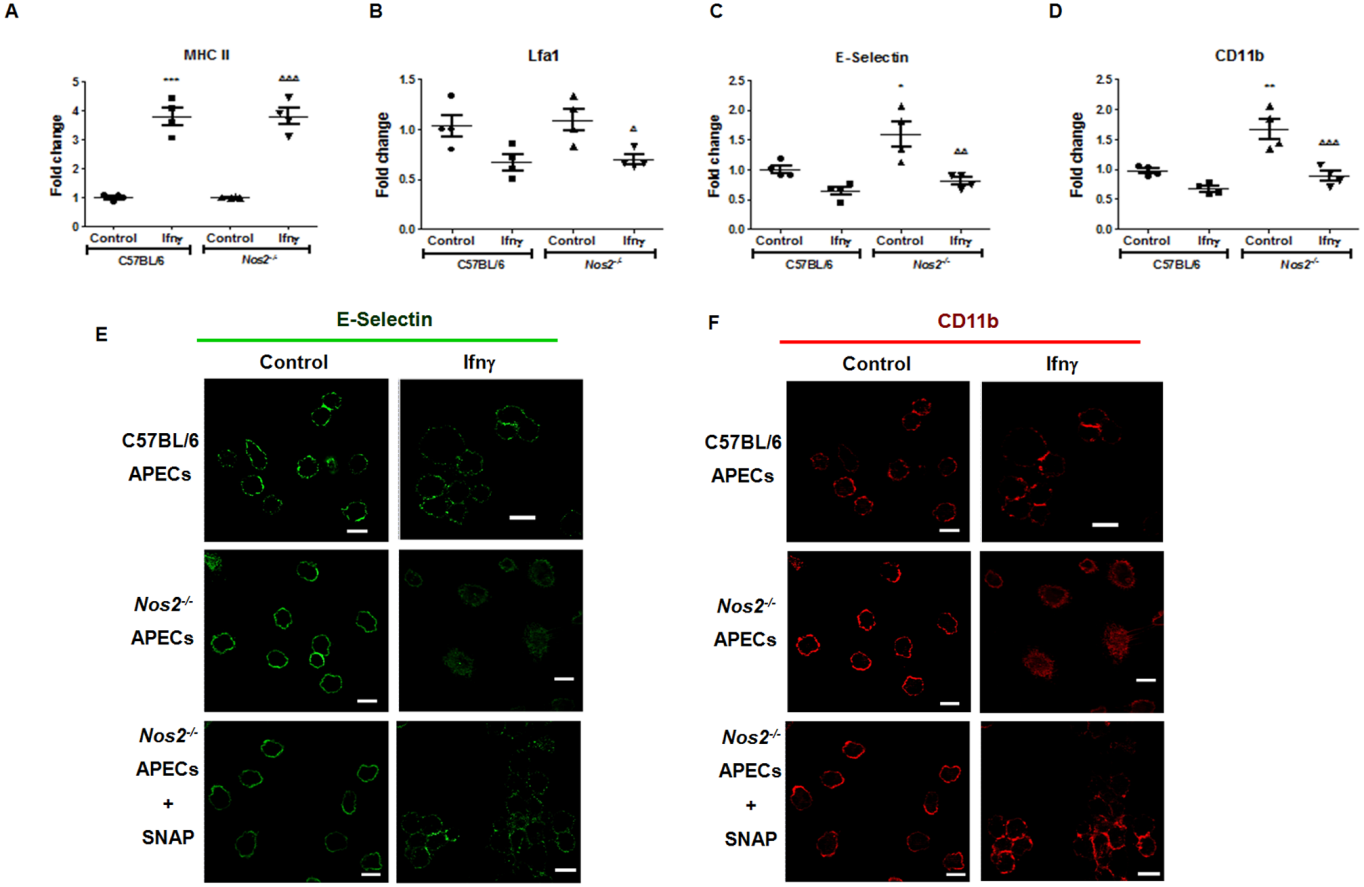
Nos2 regulates the cell surface amounts and relocalization of CD11b in response to Ifnγ in APECs. Comparative analysis of cell surface amounts of MHC class II (A), Lfa1 (B), E-Selectin (C) and CD11b (D) on APECs from C57BL/6 and *Nos2* ^*-/-*^ mice at 36 h post 25 U/ml of Ifnγ treatment. The data is represented as mean ± S.E from three independent experiments. Fluorescence microscopic images with a scale of 10 μm of E-Selectin (E) and CD11b (F) on APECs from C57BL/6 and *Nos2* ^*-/-*^ mice at 36 h post 25 U/ml of Ifnγ treatment. The significance with respect to untreated C57BL/6 APECs controls and untreated *Nos2* ^*-/-*^ APECs controls are represented as * and Δ respectively.

To investigate the roles of Nos2 in regulation of cell surface molecules by Ifnγ, further studies were performed. The induction of MHC II and repression of Lfa1 by Ifnγ were Nos2 independent (Fig 5A and 5B). Interestingly, the lack of Nos2 led to increased basal expression of CD11b as well as E-Selectin. However, Ifnγ induced repression of E-Selectin and CD11b in C57BL/6 as well as *Nos2* ^*-/-*^ APECs to a similar extent (Fig 5C and 5D). Further, fluorescence microscopic analysis revealed that lack of Nos2 led to diffused clustering and patchy cell surface localization of CD11b and E-Selectin, which was not observed on C57BL/6 APECs treated with Ifnγ (Fig 5E and 5F). Most likely, Nos2 mediates APECs aggregation, in part, by regulating the expression and cell surface localization of some cell surface molecules, e.g. CD11b, in response to Ifnγ.

### Lack of Nos2 impacts morphology of APECs

**Fig 6:**
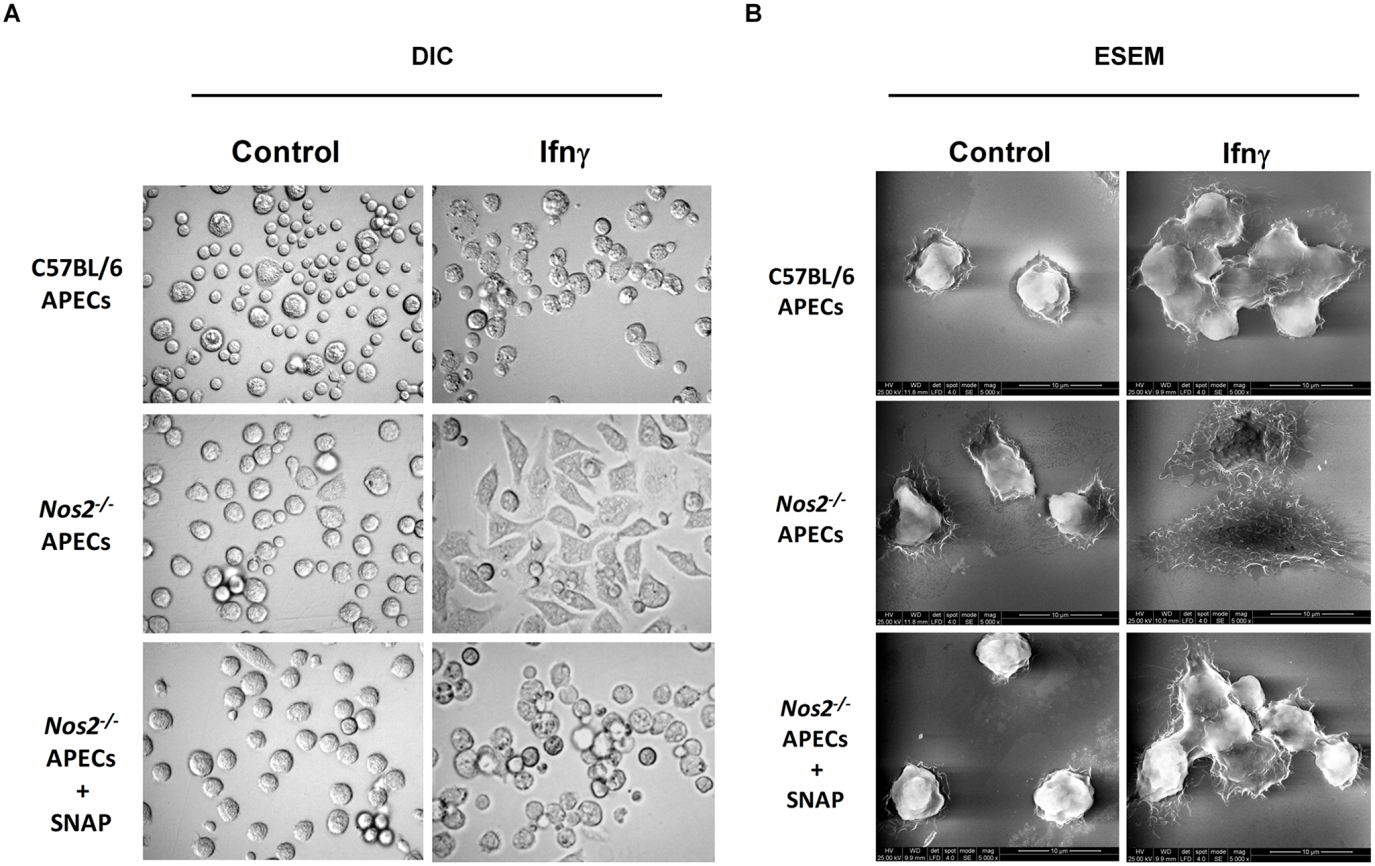
Nos2 regulates the morphology of APECs treated with Ifnγ. Representative bright field images acquired at a magnification of 60X (A) and ESEM images acquired at a magnification of 5000X (B) of C57BL/6 and *Nos2* ^*-/-*^ APECs treated with 25 U/ml of Ifnγ for 36 h is shown. In addition, the images of *Nos2* ^*-/-*^ APECs cultured in the absence or presence of 100 μM of SNAP treated in absence or presence of 25 U/ml of Ifnγ for 36 h is shown.

We observed that treatment of C57BL/6 APECs with LNMA (data not shown) or *Nos2* ^*-/-*^ APECs, in the presence of Ifnγ led to a flattened cellular morphology (Fig 6A). To understand the finer details, ESEM was performed. APECs from C57BL/6 mice appeared mostly spherical and formed stable aggregates in response to Ifnγ. On the other hand, the lack of Nos2 resulted in a highly flattened cell morphology and there was an absence of aggregate formation upon Ifnγ addition (Fig 6B). Importantly, these alterations in morphology and aggregation upon Ifnγ addition in *Nos2* ^*-/-*^ APECs could be functionally reversed by adding SNAP exogenously, demonstrating NO dependence (Fig 6A and 6B). These data highlight the requirement of Nos2 derived NO in conjunction with other Ifnγ signals in mediating aggregate formation as well as maintenance of morphology in APECs.

### Absence of Nos2 does not skew macrophage polarization signals in response to Ifnγ

Ifnγ in known to polarize macrophages to the M1 phenotype and the induction of Nos2 is an important marker [26]. Therefore, we investigated whether the effects observed in terms of morphology, motility and aggregation in *Nos2* ^*-/-*^ APECs were due to any shift in macrophage polarizing signals. To test this hypothesis, several known markers for M1 and M2 identity were studied [26,27]. Ifnγ treatment led to increased cell surface MHC I and CD80, but not CD86, levels to a similar extent in both C57BL/6 and *Nos2* ^*-/-*^ APECs (Fig. C in S1 File). Also, Ifnγ treatment reduced basal Arginase1 levels to a similar extent in C57BL/6 and *Nos2* ^-/-^ APECs (Fig. C in S1 File). The analysis of cell-free supernatants revealed that Tumor Necrosis Factor-α (Tnfα), but not Il10, (data not shown) amounts increased in response to Ifnγ treatment in C57BL/6 as well as *Nos2* ^*-/-*^ APECs. Overall, the lack of Nos2 in APECs does not affect the markers of M1 polarization in response to Ifnγ.

### Nos2 regulates cytoskeleton elements, Actin and Tubulin, in response to Ifnγ

Macrophages are known to be motile and professional antigen presenting cells. To understand whether the addition of Ifnγ affected motility and the possible contributions of Nos2 to this process, live cell imaging studies were performed. Tracking of cells as a function of time using live cell imaging analysis led to two interesting and novel observations: first, C57BL/6 APECs exhibited a basal and random motility that was reduced by the treatment of Ifnγ (Fig. D in S1 File and S1 and S2 Videos). Second, the lack of Nos2 reduced basal motility of APECs and these cells flattened upon Ifnγ treatment compared to untreated C57BL/6 controls (Fig. D in S1 File and S3 and S4 Videos). Clearly, these data demonstrate that Nos2 is important for basal motility and the maintenance of morphology in response to Ifnγ treatment in APECs.

**Fig 7:**
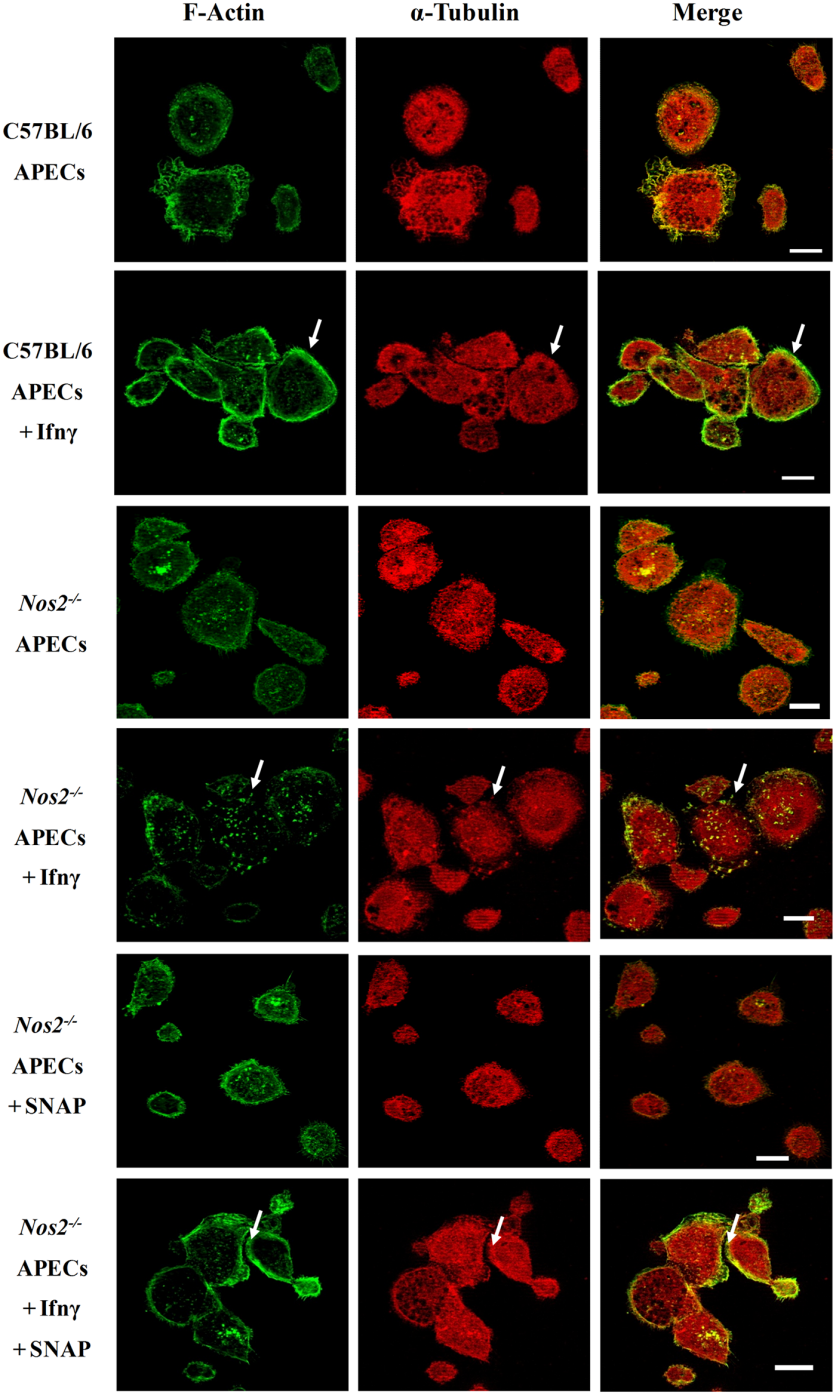
The cortical stability of cytoskeleton elements, Actin and Tubulin, is regulated in a Nos2 dependent manner upon Ifnγ treatment. Fluorescence microscopic images, with the scale bar of 10 μm, of F-Actin (green) and α-Tubulin (red) in APECs from C57BL/6 mice and *Nos2* ^*-/-*^ mice treated without or with 25 U/ml of Ifnγ for 36 h. In addition, *Nos2* ^*-/-*^ APECs were treated with 100 μM of SNAP in the absence or presence of Ifnγ. The white arrows indicate cortical arrangement of F-Actin and α-Tubulin.

The above results led us to examine the possible regulation and roles of two important cytoskeleton proteins, Actin and Tubulin. In C57BL/6 APECs, addition of Ifnγ led to stabilization of cortical F-Actin. In case of α-Tubulin, it was found evenly spread in the cytoplasm. Importantly, in the absence of Nos2, addition of Ifnγ led to displacement of the cortical Actin and Tubulin network. This defect in *Nos2* ^*-/-*^ APECs was not reversed by the exogenous addition of SNAP alone. However, the combination of SNAP with Ifnγ led to restoration of the cortical arrangement of Actin and Tubulin in *Nos2* ^*-/-*^ APECs (Fig 7). This aspect of regulation of cortical stability of network of Actin and Tubulin by Nos2 in APECs, only in the presence of Ifnγ has been quantified and represented in Fig. E in S1 File.

### Actin and Tubulin stability contribute to motility, morphology and aggregation of APECs

**Fig 8:**
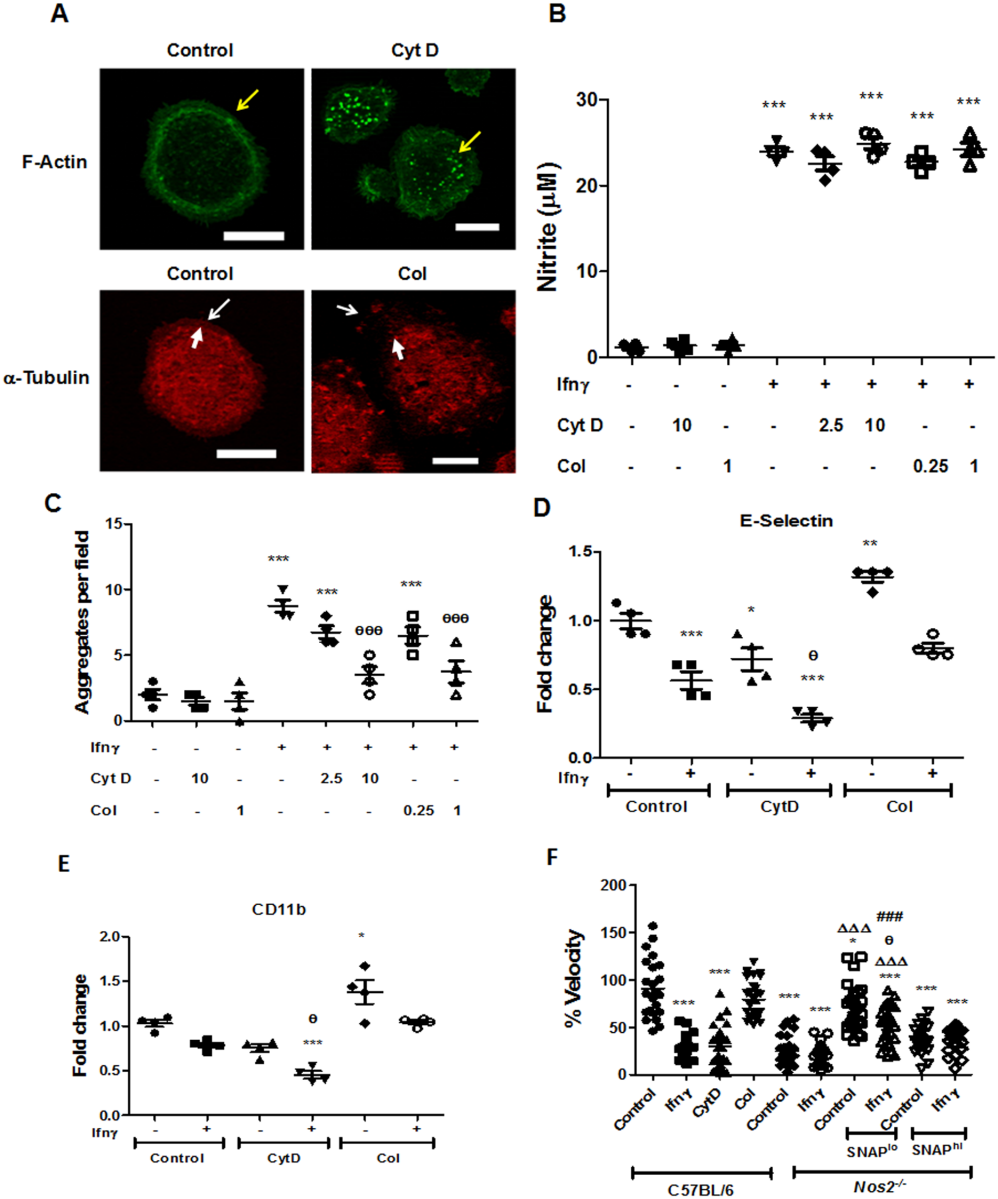
Actin and Tubulin stabilization contribute to Ifnγ induced aggregation of APECs. Fluorescence microscopic images acquired at a magnification of 63X with the scale bar representing 10 μm of F-Actin (green) and α-Tubulin (red) in C57BL/6 APECs treated with 10 μM Cyt D and 1 μg/ml of Col respectively for 6 h (A). Yellow and white arrows indicate cortical arrangement of F-Actin and α-Tubulin respectively. Thin white arrows indicate the cell boundary while the fatter white arrows are used to highlight the shrinkage of the α-Tubulin network upon Col treatment when compared to the untreated control C57BL/6 APECs. The amount of nitrite in the supernatant (B) produced by APECs from C57BL/6 mice upon treatment with 25 U/ml of Ifnγ in the absence or presence indicated doses of inhibitors Cyt D (μM) and Col (μg/ml). The number of cell aggregates (C) of APECs from C57BL/6 mice upon treatment with 25 U/ml of Ifnγ in the absence or presence of indicated doses of inhibitors Cyt D (μM) and Col (μg/ml). The amounts of E-Selectin (D) and CD11b (E) on the cell surface of C57BL/6 APECs treated with 25 U/ml of Ifnγ for 36 h, post 6 h of pretreatment without or with Cyt D (10 μM) and Col (1 μg/ml). The data is represented as mean ± S.E from three independent experiments. The velocity of APECs from C57BL/6 and *Nos2* ^*-/-*^ mice treated without or with 25 U/ml of Ifnγ between 18–24 h of addition (F). The velocity of APECs from C57BL/6 pretreated for 6 h with Cyt D (10 μM) and Col (1 μg/ml) before tracking them from 6–12 h post treatment. Also, the velocity of *Nos2* ^*-/-*^ APECs pretreated in the presence of 5 μM of SNAP (SNAP^lo^) and 100 μM of SNAP (SNAP^hi^) for 12 h and without or with 25 U/ml of Ifnγ before tracking cells 18–24 h post Ifnγ addition. The velocity for each of the above mentioned conditions are represented as percentage velocity with respect to average velocity exhibited by untreated C57BL/6 APECs. The data is representative of two independent experiments. Significance is represented as * when compared to untreated controls and # when compared to Ifnγ treated C57BL/6 APECs respectively. The significance with respect to untreated controls, Ifnγ treated C57BL/6 APECs controls. untreated *Nos2* ^*-/-*^ and SNAP^lo^ alone *Nos2* ^*-/-*^ APECs controls are represented as *, θ, Δ, and # respectively.

To “directly” assess the roles of stabilization of Actin and Tubulin during the aggregation of APECs, studies were performed with Cyt D, a potent Actin depolymerizing compound, and Col, a potent microtubule depolymerizing agent. First, Cyt D and Col treatment led to Actin depolymerization and shrinkage of the Tubulin network as revealed by their retraction from the cell membrane in C57BL/6 APECs (Fig 8A). These inhibitors did not affect the Ifnγ induced nitrite production by APECs (Fig 8B), confirming our observations that the Actin and Tubulin stabilization events were downstream to Nos2 induction. Addition of either of these inhibitors, in a dose dependent manner, led to significant reduction in the formation of aggregates in response to Ifnγ (Fig 8C). Interestingly, addition of Cyt D reduced, whereas Col addition increased, basal amounts of E-Selectin and CD11b levels on the cell surface without affecting their fold repression upon Ifnγ addition (Fig 8D and 8E). Live cell imaging analysis revealed that addition of Cyt D, but not Col, reduced basal motility of APECs (Fig 8F, Fig. D in S1 File, S1 and S5 Videos). However, addition of Col, but not Cyt D, led to flattened morphology of APECs, similar to those seen in *Nos2* ^*-/-*^ APECs treated with Ifnγ (S6 Video). Most likely, Actin stabilization contributed to the motility, whereas Tubulin stabilization was important for the maintenance of morphology in APECs. Both Actin and Tubulin were regulated by Nos2 generated NO in response to Ifnγ treatment in these cells.

To address the roles of the extent of NO in affecting motility of APECs, experiments with low and high dose of SNAP were performed. Live cell imaging analysis revealed that addition of Ifnγ or CytD, but not Col, reduced the basal motility and velocity of C57BL/6 APECs. Also, the lack of Nos2 reduced basal motility and velocity when compared to C57BL/6 APECs (Fig 8F, Fig. D in S1 File and S1–S6 Videos). Interestingly, addition of low dose of SNAP (lo) to *Nos2* ^*-/-*^ APECs significantly increased basal motility and velocity to that of C57BL/6 APECs controls. Importantly, addition of higher dose of SNAP (hi) lowered basal motility and velocity in *Nos2* ^*-/-*^ APECs (Fig 8F and Fig. D in S1 File). It is likely that there are thresholds for the effects of NO on Actin and Tubulin that may be important in determining the extent of motility of APECs.

As macrophages are professional phagocytes, the possible contributions of Nos2, Actin and Tubulin to the phagocytic ability of APECs was evaluated using the internalization of latex beads. The phagocytic ability of C57BL/6 APECs was reduced with LNMA and CytD, but not Col, treatment (Fig. F in S1 File). Also, loss of Nos2 decreased the phagocytic ability of APECs considerably (Fig. F in S1 File). Further confirmatory experiments were performed using APECs from C57BL/6 and *Nos2* ^*-/-*^ mice that were infected with *S*. Typhimurium and their internalization, i.e entry into cells, was monitored after 2 h post infection. As seen in Fig. F in S1 File, the CFU recovered from *Nos2* ^*-/-*^ APECs were significantly reduced in comparison to their C57BL/6 counterparts. Overall, the amounts of Nos2 generated NO regulate several functions of APECs: motility, phagocytosis, morphology and aggregation.

### APECs from *S*. Typhimurium infected mice aggregate in a Nos2 dependent manner

**Fig 9:**
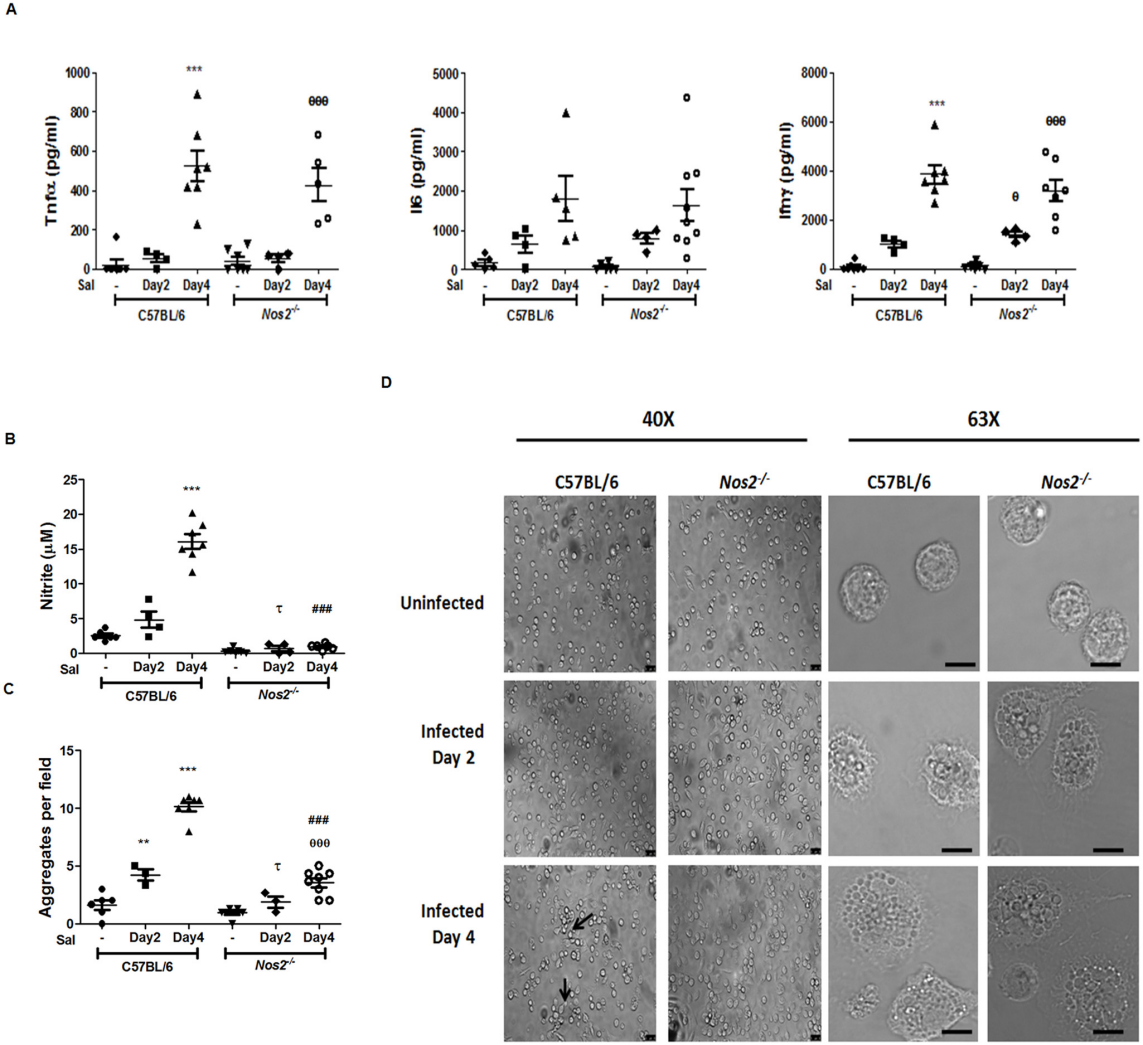
Nos2 mediates aggregation of APECs upon *S*. Typhimurium infection of mice. The amounts of Tnfα, Il6 and Ifnγ (A) in the sera from uninfected and *S*. Typhimurium infected C57BL/6 and *Nos2* ^*-/-*^ mice at day two and four post infection. The amounts of nitrite (B) and cell aggregates (C) of APECs isolated from uninfected and *S*. Typhimurium infected C57BL/6 and *Nos2* ^*-/-*^ mice at indicated days post infection and cultured *ex vivo* for 24 h. Representative bright field microscopic images of APECs, either at 40X (Leica DMI6000B) or 63X (Leica TCS SP5), with the scale bar representing 20 μm, of APECs from uninfected or infected mice are shown (D). The data is representative of at least four independent experiments with a minimum of three mice per condition. Significance with respect to untreated C57BL/6 controls, untreated *Nos2* ^*-/-*^ controls, *S*. Typhimurium infected C57BL/6 mice day 2 control and *S*. Typhimurium infected C57BL/6 mice day 4 control are represented as *, θ, τ, and # respectively.

**Fig 10:**
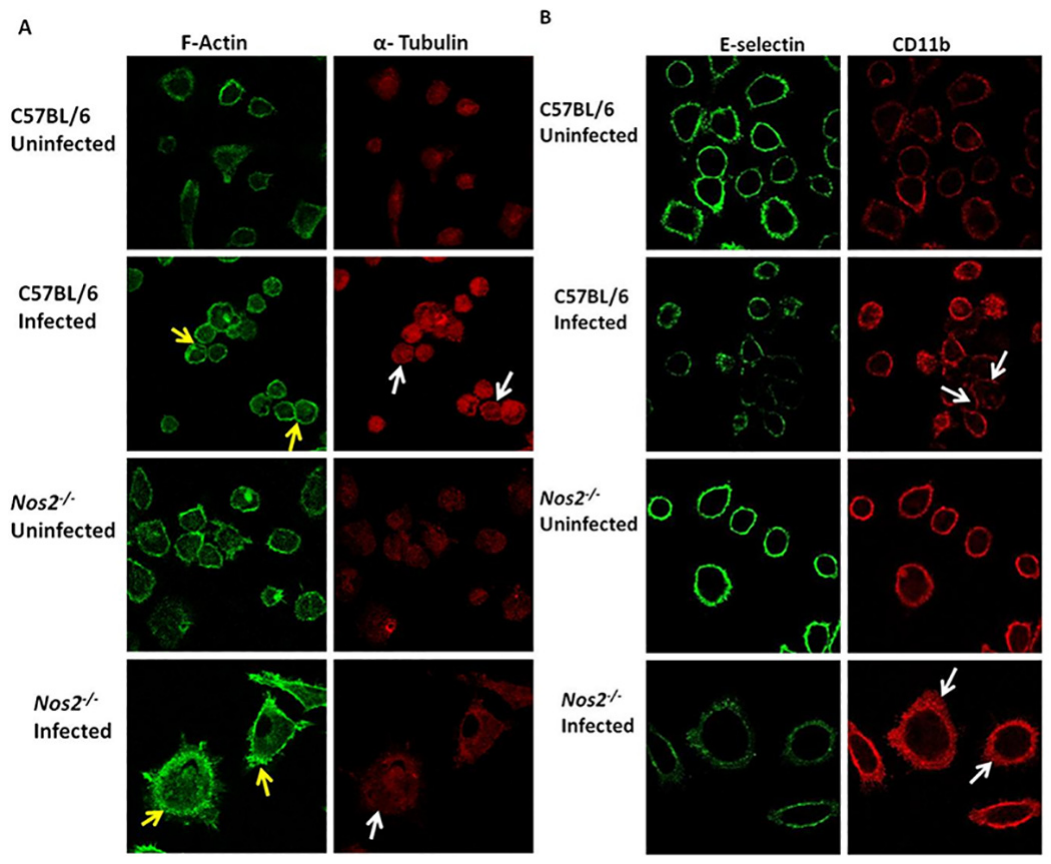
Differences in localization patterns of Actin, Tubulin, CD11b and E-Selectin in APECs from C57BL/6 and *Nos2* ^*-/-*^ APECs upon infection. Fluorescence microscopic images of F-Actin (green) and α-Tubulin (red) in APECs from uninfected and *S*. Typhimurium infected C57BL/6 and *Nos2* ^*-/-*^ mice four days post infection, left untreated *ex vivo* for 24 h (A). Yellow and white arrows represent Actin and Tubulin localization to the cortex, respectively. Confocal microscopic images of E-Selectin (green) and CD11b (red) in APECs taken at 100X magnification from uninfected and *S*. Typhimurium infected C57BL/6 and *Nos2* ^*-/-*^ mice four days post infection, left untreated *ex vivo* for 24 h (B). The white arrows depict CD11b localization at the cell-cell interface in C57BL/6 APECs from infected mice. On the other hand, APECs from infected *Nos2* ^*-/-*^ mice display diffused CD11b pattern. Scale bar for fluorescent microscopic images is 10 μm. The data is presented as mean ± S.E from two independent representative experiments with three mice per group.

It was important to address the biological relevance of Nos2-mediated modulation of functional responses in APECs. The approach utilized was to study the response of APECs from C57BL/6 and *Nos2* ^-/-^ mice post oral infection with *S*. Typhimurium. The amounts of cytokines, Tnfα Interleukin 6 (Il6) and Ifnγ in the sera increased significantly by day 4 post infection in both C57BL/6 and *Nos2* ^*-/-*^ mice (Fig 9A). Also, there was no major difference in the population of APECs obtained from uninfected and upon *S*. Typhimurium infection of mice (Fig. G in S1 File). The amounts of nitrite produced by APECs from uninfected mice, in the absence of any activating signals, was low. However, high amounts of nitrite were detected in the supernatants of C57BL/6 APECs, but not in their *Nos2* ^*-/-*^ counterparts, four days after infection (Fig 9B). Importantly, APECs from infected C57BL/6 mice aggregated by 24 h upon *ex vivo* culture, whereas the APECs from *Nos2* ^*-/-*^ mice were flattened and formed significantly lesser number of aggregates (Fig 9C and 9D). Also, APECS from both C57BL/6 and *Nos2* ^-/-^ infected mice were slightly larger and displayed a granular and vacuolated morphology compared to uninfected C57BL/6 mice (Fig 9D). Further, prominent cortical arrangement of F-Actin and α-Tubulin was observed in APECs from infected C57BL/6 mice. However, in APECs from infected *Nos2* ^*-/-*^ mice, there was reduction in the cortical localization of Actin compared to their C57BL/6 counterparts (Fig 10A). Also, both E-Selectin and CD11b were found to be expressed on the cell surface, with CD11b re-localizing to the sites of interaction between APECs, from infected C57BL/6 mice. APECs from infected *Nos2* ^*-/-*^ mice did not aggregate and the localization of both E-Selectin and CD11b was found to be more diffused (Fig 10B). Thus, *Nos2*-dependence of the aggregation responses of APECs was also observed during an inflammatory model of *S*. Typhimurium infection in mice.

### APECs in aggregates are more efficient in reducing the number of intracellular *S*. Typhimurium

**Fig 11:**
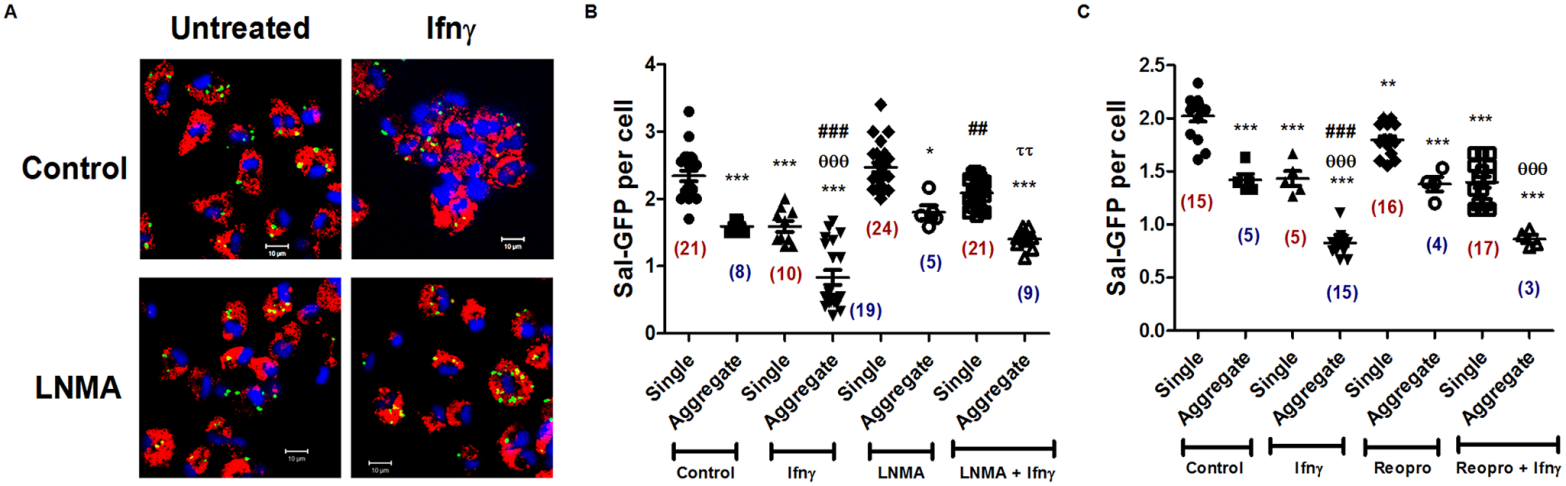
The aggregation of APECs reduces the intracellular growth *S*. Typhimurium. Representative confocal microscopic images acquired at a magnification of 100 X with a scale of 10 μm illustrating intracellular Sal-GFP (green), Lamp1 (red) and Hoechst (blue) in control and 25 U/ml of Ifnγ treated APECs in the presence or absence of LNMA (200 μM) post 24 h of Sal-GFP infection (A). Quantification of the number of Sal-GFP per cell in control and 25 U/ml of Ifnγ treated APECs in the presence or absence of LNMA (200 μM) post 24 h of Sal-GFP infection (B). Quantification of the number of Sal-GFP per cell in control and 25 U/ml of Ifnγ treated APECs in the presence or absence of Reopro (0.2 mg/ ml) post 24 h of Sal-GFP infection (C). In panel B and C, the number of fields scored for single cells are represented in maroon color and the number of fields scored with aggregates are represented in blue color within brackets under the data points. Note that in the presence of Ifnγ, the number of aggregates containing fields increase compared to fields with single cells, which is reversed with LNMA or Reopro treatment. The data is represented as mean ± S.E from two independent experiments. Significance is represented as * when compared to untreated single cell controls, # when compared to Ifnγ treated single cell controls and Δ when compared to Ifnγ treated aggregates of APECs controls. The significance is represented as * when compared to untreated single cell controls, θ when compared to untreated aggregate controls, # when compared to Ifnγ treated single cell controls and τ when compared to Ifnγ treated aggregates of APECs controls.

Does the aggregation of APECs constitute an advantage for the host defense network? To address this question, the ability of single and aggregated APECs to control the number of intracellular *S*. Typhimurium expressing green fluorescent protein (Sal-GFP) was studied *in vitro*. APECs from uninfected mice were isolated and an MOI of 1:50 was selected based on titration experiments (Fig. F in S1 File). Subsequently, APECs from uninfected mice were isolated, Ifnγ was added 2 h post infection and cells were imaged at 24 h post Ifnγ treatment. Upon infecting C57BL/6 APECs, fewer Sal-GFP were found in cells that were in aggregates as compared to cells that were single (Fig 11A). It should be pointed out that the number of such aggregates were low in the absence of Ifnγ stimulation. Upon addition of Ifnγ, some decrease (~32%) in the number of Sal-GFP was observed in cells that remained single; importantly, significant decrease (~50%) in Sal-GFP was observed in aggregates (Fig 11A–11C).

To study whether blocking the aggregation of APECs had any impact on the ability to lower intracellular *S*. Typhimurium numbers, cells were treated with LNMA 2 h post infection (to avoid any effects on bacterial internalization into host cells) in the presence or absence of Ifnγ. In the absence of Ifnγ, the addition of LNMA did not significantly affect Sal-GFP numbers. In the presence of Ifnγ, addition of LNMA increased the number of APECs that were single and these demonstrated higher intracellular Sal-GFP (~16% drop as compared to ~32% in Ifnγ alone controls). Also, as seen in (Fig 11A and 11B), the few aggregates of APECs that were present upon LNMA and Ifnγ co-treatment had significantly higher Sal-GFP (~24% drop as compared to ~50% in Ifnγ alone controls) upon Ifnγ treatment in comparison to Ifnγ alone counterparts. Hence, NO contributed significantly to the formation of aggregates, which were better equipped to clear infection by the intracellular pathogen *S*. Typhimurium. To delineate the role of aggregation from the bactericidal functions of NO, experiments with Reopro were performed to address the role of CD11b in cell-cell interactions. Addition of Reopro led to a marginal decrease in the number of Sal-GFP per cell. In the presence of Ifnγ, Reopro did not significantly impact Sal-GFP per cell in either singles (~24% drop as compared to ~25% in Ifnγ controls) or in aggregates (~39% drop as compared to ~40% in Ifnγ controls) of APECs (Fig 11C). Importantly, Reopro treatment decreased the ability of APECs to form aggregates and, as a consequence, increased the number of APECs that remained single. Consequently, these single APECs were unable to resist infection as much as the aggregated APECs (Figs 3D and 11C). These experiments delineated the distinct roles of NO and CD11b: CD11b promoted aggregation of APECs whereas NO contributed to aggregation of APECs and lowered intracellular bacterial replication.

## Discussion

**Fig 12:**
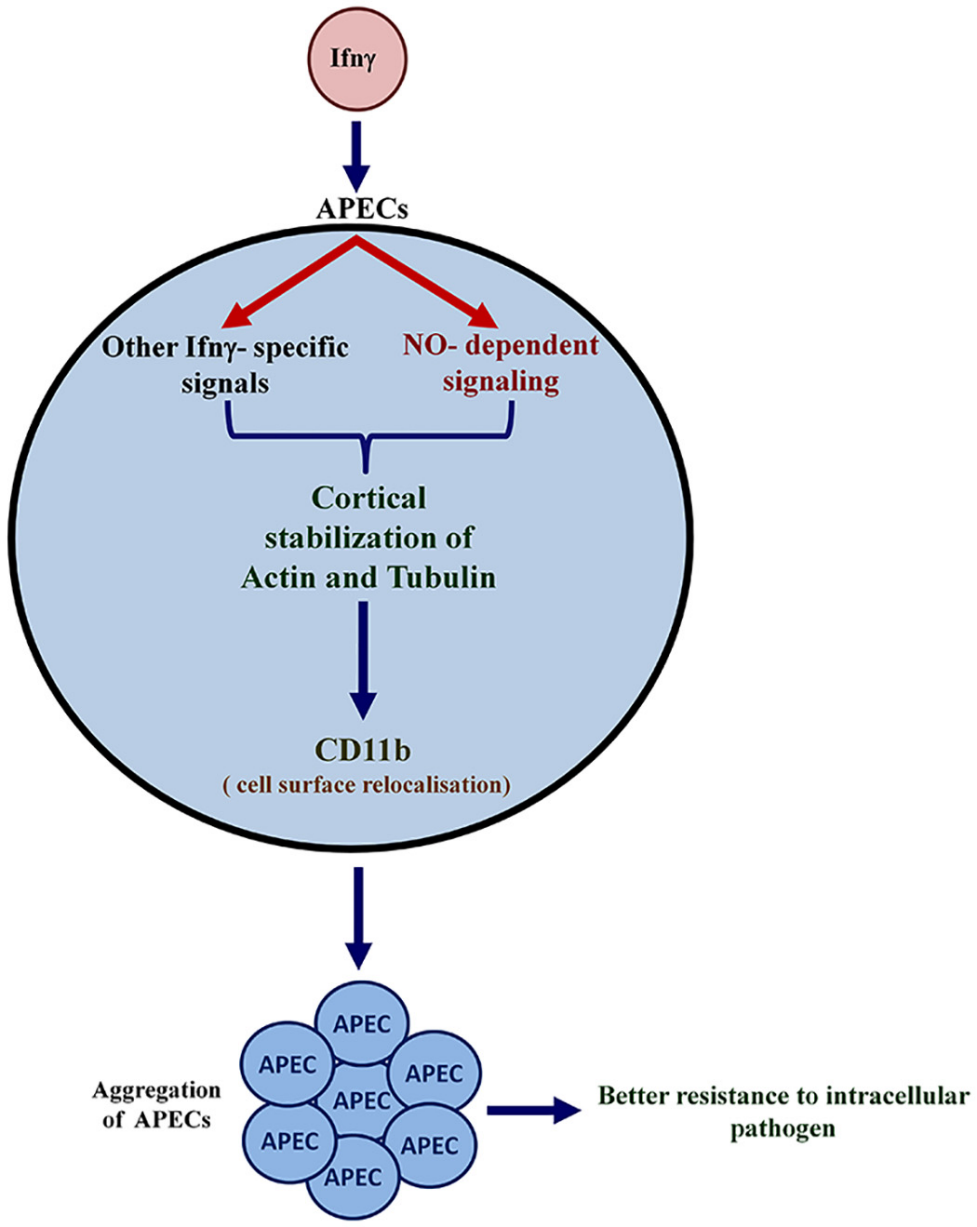
A schematic model representing the aggregation response of APECs in response to Ifnγ treatment. In the presence of Ifnγ, Nos2 is induced resulting in high amounts of NO. Both NO and additional Ifnγ specific signals are required for stability and cortical arrangement of Actin and Tubulin, which affects several cellular functions, including motility, phagocytosis and maintenance of morphology. In addition, re-localization of CD11b enhances the aggregation of APECs which are more efficient in reducing the number of intracellular bacteria and increasing host resistance.

There have been several efforts to understand the mechanisms and functional consequences of interactions between different cells of the immune system. The ability of neutrophils to swarm to the site of wound and form aggregates is dependent on Leukotriene B4 and integrins [28]. However, the ability of APECs consisting primarily of macrophages to interact with each other, the mechanisms involved and the possible physiological significance have not been explored. In this study, we demonstrate that Ifnγ specifically induces APECs to form aggregates (Fig 1). Two important mediators of the responses to Ifnγ are ROS and NO [19–21]. The role of ROS in contributing to platelet aggregation, for example, has been well documented [29]. However, Ifnγ induced ROS played a negligible role during aggregation of APECs. On the other hand, platelet aggregation is inhibited by NO generated by both platelets and endothelial cells [29]. Also, NO generated in response to Ifnγ inhibits T cell adhesion to the endothelium in the presence of Tnfα [30]. Our data establishes the roles of NO using a chemical inhibitor, e.g. LNMA, and cells from mice lacking Nos2 during Ifnγ induced aggregation of APECs (Fig 4). We have also shown that Ifnγ and Nos2-derived NO is important for motility, phagocytosis and morphology in APECs. It needs to be highlighted that addition of SNAP or DETA/NO, two distinct NO donors, alone was insufficient to induce formation of aggregates in APECs. Hence, NO produced in conjunction with other intracellular Ifnγ signals contributes to the formation of aggregates in APECs (Fig 12).

It is pertinent to discuss some aspects related to this study: First, the aggregation of APECs in response to Ifnγ was specific to these cells and was not seen with bone marrow derived macrophages (data not shown). This aspect is relevant as tissue-resident macrophages display high transcriptional and functional diversity. Tissue-resident macrophages are seeded very early during development and their numbers are self-maintained through adult life with a minimal contribution from circulating monocytes [5,31]. Second, resident APECs are known to respond well to Ifnγ, but much lower to LPS, in terms of nitrite production [32]. We found that LPS, another inflammatory stimuli, failed to induce aggregation of APECs (data not shown), demonstrating that the effect was specific to Ifnγ. In fact, the ability of Ifnγ, but not LPS, to lower the amounts of Ccl3 and Ccl4 is also dependent on the activation status of macrophages [19]. Most likely, the tissue origin and activation status are important factors in modulation of some responses by resident peritoneal macrophages. Third, to demonstrate the direct effects of NO, two NO donors were used, which rescued several alterations observed in *Nos2* ^-/-^ APECs upon Ifnγ treatment. However, a couple of caveats need to be kept in mind: first, the amounts of SNAP and DETA/NO used were similar to the amounts of Ifnγ-induced nitrite. Second, the half life of SNAP is shorter compared to DETA/NO [25]; however, the effects obtained with Ifnγ were similar (Figs 4–8 & Fig. B in S1 File). Most likely, sustained NO release may not be required as the amount of NO produced initially is sufficient to initiate signaling events leading to the rescue in phenotype observed in *Nos2* ^-/-^ APECs with Ifnγ.

Fourth, Ifnγ-induced NO is known to be cytotoxic to some cells [20,21]; however, the viability of APECs in presence of Ifnγ from C57BL/6 and *Nos2* ^-/-^ was not affected (Fig 4G). Hence, it is unlikely that the morphology and other changes observed in this study are due to cells undergoing death. Fifth, macrophages are plastic and highly responsive to environmental stimuli [26,27]. In fact, a recent report has shown that change in the shape of macrophages affects their ability to polarise [33]. Therefore, we investigated whether the responses observed could be due to the polarization of *Nos2* ^-/-^ APECs towards the M2 phenotype. Ifnγ is known to reduce Arginase amounts, an important M2 polarization factor [34] and reduction of Arginase was observed in C57BL/6 and *Nos2* ^-/-^ APECs to a similar extent (Fig. C in S1 file). There was no major difference in the expression of the panel of phenotypic markers in response to Ifnγ in C57BL/6 and *Nos2* ^-/-^ APECs (Fig. C in S1 File). Also, Ifnγ induction of Cxcl10, a M1 marker [26], by APECs is Nos2 independent [19]. There was no difference between C57BL/6 and *Nos2* ^-/-^ APECs with respect to M1 polarization characteristics. Thus, Nos2 regulates some key, but not all, responses of APECs.

Several studies have demonstrated the association of Nos2 with cytoskeletal elements which is required for optimal function of the enzyme [35]. Both Actin and Tubulin are nitrosylated but there are differential reports on the direct versus indirect effects of nitrosylation and downstream functional consequences. S-nitrosylation of four cysteines at the carboxyl terminus of Actin in neutrophils alters Actin polymerization and intracellular distribution [36]. On the other hand, NO induces ADP-riboyslation of Actin, which inhibits its polymerization; consequently, phagocytosis and some other functions of macrophages are affected [37]. Another study identified that the NO mediated regulation of Actin is via cGMP and Ca^2+^/Calmodulin [38]. Nitrosylation of Actin by peroxynitrite, a potent oxidant, inhibits several cellular processes of neutrophils such as migration, phagocytosis and respiratory burst [39]. NO affects the motility and shape of bone marrow derived stromal cells [40], and signaling proteins, such as the Protein kinase 2, induce directional migration and maintain morphology of macrophages [41]. Also, nitrosylation of Tubulin is important as it leads to redistribution of cellular proteins, alters cell morphology etc [42]. In this study, we show that the cortical stabilization of Actin and Tubulin by Ifnγ is affected in a Nos2 dependent manner (Fig 7). Differences are observed in Actin and Tubulin in human macrophages post treatment with depolymerizing compounds [43]. In fact, these changes in Actin and Tubulin were also observed when APECs were treated with depolymerizing compounds (Fig 8A). Both Actin and Tubulin stabilization contributed to Ifnγ induced aggregation of APECs (Fig 8C). Strikingly, APECs lacking Nos2 resembled wild type cells treated with the Actin depolymerizing compound in terms of basal motility (Fig 8F and Fig. D in S1 File). Moreover, APECs from *Nos2* ^*-/-*^ mice were flattened in response to Ifnγ treatment; notably, these cells resembled wild type APECs treated with Col (S4 and S6 Videos) or LNMA (data not shown). Hence, the observation that Ifnγ induced excess production of NO lowers motility (Fig 8F) is consistent with the available literature. However, the observation that lack of endogenous Nos2 derived NO greatly lowers basal motility (Fig 8F) and phagocytic activity (Fig. F in S1 File) is novel. Together, the data suggests the following model: Ifnγ and NO stabilize cortical Actin and Tubulin which are important for some functions in APECs (Fig 12).

Leukocytes are known to express enhanced amounts of several β-2 integrins, which are critical for them to extravasate into inflamed tissue. In fact, patients lacking β-2 integrin succumb to bacterial and other infections early in their life as a consequence of ‘Leukocyte adhesion deficiency’ [44]. The roles of CD11b in promoting neutrophil infiltration and resistance to systemic lupus erythematoses are well established [45,46]. In this study, CD11b localized to the sites of interaction and functionally contributed to APEC aggregation in response to Ifnγ in a Nos2 dependent manner. A study has shown Nos2 to be associated with F-Actin via Focal adhesion kinase. Upon nitrosylation of Actin, its interaction with Focal Adhesion kinase is reduced which lowers β-integrin function and inhibition of neutrophil adhesion to endothelial cells [47]. The observation that the basal cell surface expression of CD11b and E-Selectin, but not Lfa1, was higher in *Nos2* ^-/-^ mice is interesting. NO is known to modulate the expression of some cell surface molecules using different mechanisms, e.g. actin-focal adhesion kinase [47] or cGMP-Protein kinase G [48]. In this study, NO has been shown to affect cytoskeletal elements and these changes may affect the basal expression of some cell surface proteins. Indeed, cytoskeleton denaturing compounds modulate the expression of some cell surface molecules [49,50]. Most likely, the induction of NO with Ifnγ stabilizes cortical arrangement of Actin and Tubulin, which contribute to slightly lowered amounts and cell surface relocalization of CD11b (Fig 12). Further studies are required to evaluate the expression and functional roles of additional cell surface molecules that may be involved during the Ifnγ-mediated aggregation of APECs.

The recruitment of macrophages to the site of wound and inflammation is finely orchestrated. In an *in vivo* model of sterile skin injury, macrophages arrive late to the site and adhere to the formed neutrophil clusters [28]. To understand whether Nos2 impacted signals for APECs to aggregate, oral infection of mice with *S*. Typhimurium was performed. Most likely, the high amounts of inflammatory cytokines on day 4 post infection led to increased production of nitrite observed by C57BL/6 APECs from infected mice (Fig 9). Upon incubation of cells *ex vivo* from the infected mice, clear differences in the aggregation capabilities were observed in APECs from infected C57BL/6 and *Nos2* ^*-/-*^ mice recapitulating the observations in the *in vitro* model of Ifnγ induced APEC responses (Fig 10). *S*. Typhimurium is known to prevent Nos2 containing vacuoles from fusing with *Salmonella* containing vacuoles using the SPI-2 mediated Type III secretion system [51]. Also, Nos2 localizes along with cortical actin in macrophages; however, it does not get recruited to phagosomes and *Salmonella* containing vacuoles [52]. In order to affect antibacterial responses it is possible that Nos2 regulates host processes other than direct delivery of bactericidal NO to the location of target. Indeed, a recent study has shown that Nos2 increases the expression of an iron exporter, Ferroportin-1, which is responsible for lowering the intracellular iron requirement for optimal growth of *S*. Typhimurium in macrophages [53]. Although Ifnγ lowered the replication of *S*. Typhimurium in both single and aggregated cells, the fold drop was greater in the latter. The individual roles of NO, critical for aggregate formation and bactericidal activity (either directly or indirectly), and CD11b, important for aggregation of APECs, were dissected (Fig 11). The possibility that the presence of cellular aggregates, in contrast to APECs that are single, facilitates higher local concentration of antibacterial agents, including ROS, NO, antibacterial peptides etc is enticing and warrants further investigation.

It may be worthwhile to discuss the relevance of this study in human cells. In general, human macrophages do not produce high amounts of NO in response to inflammatory stimuli. A recent study has shown the presence of methylated CpG motifs proximal to the *NOS2* transcription site and a closed chromatin state in human macrophages. This is in contrast to the unmethylated motifs and open chromatin state present in mouse macrophages [54]. However, NO is generated by human cells under some conditions, e.g. infection with *Mycobacterium tuberculosis* [55] or respiratory syncicial virus [56] or during chronic obstructive pulmonary disease [57] etc. Human macrophages are also known to produce NO in response to *Salmonella* infection. In this context, it is interesting that *Salmonella* encode proteins that protect them against host encoded NO. Indeed, a *S*. Typhimurium strain lacking flavohemoglobin is highly sensitive to NO [58]. Further studies will be required to evaluate the relevance of our observations in human cells under different disease and inflammatory conditions.

Mice lacking Nos2 were generated nineteen years ago [59] and several studies have revealed the importance of this enzyme in regulating a wide variety of immune responses [60]. The bactericidal role of NO against a plethora of intracellular pathogens is well appreciated [24,60]. Our observations are novel and the two major highlights of the present study are: First, several Ifnγ-mediated responses by APECs, i.e. motility, phagocytosis, maintenance of morphology and aggregation, are dependent on Nos2. Second, aggregation or group behavior of APECs is important for effective resistance against intracellular pathogens, e.g. *S*. Typhimurum. In addition to their known abilities to produce specific antimicrobial compounds, it is possible that innate immune cells such as APECs interact and function as a group to effectively combat intracellular pathogens. This ability of Nos2 generated NO to form aggregates of APECs and function as a group might help the immune system to perform newer and enhanced functions in contrast to cells that are single. In future, a combination of genetics and sophisticated imaging techniques needs to be employed to investigate aggregation of APECs *in vivo*. Further work is required to understand the possible causes and functional relevance of cellular aggregation during homeostatic and diseased states in greater detail. Also, Nos2 dependent *in vivo* group interactions between macrophages and with other immune cell types may be important during homeostasis and responses to immune challenge. Therefore, a better understanding of the roles of molecules important for macrophage interactions and functions may lead to development of therapeutic compounds that influence immune and inflammatory outcomes in favor of the host.

## Supporting Information

S1 File **Fig. A in S1 File. Transfection with scrambled siRNA does not alter major responses of APECs.** APECs from C57BL/6 with or without transfection with 250nM scrambled siRNA were treated in the presence or absence of 25U/ml of Ifnγ for 24h. The amounts of nitrite (A), Fold change in cell surface CD11b expression (B) and number of cell aggregates per field (C) were quantified. The data is representative of at least three different mice in each condition. The significance with respect to untransfected control are represented as *. **Fig. B in S1 File. DETA/NO induces aggregation in *Nos2*** ^***-/-***^ **APECs upon Ifnγ treatment.** APECs from C57BL/6 and *Nos2* ^*-/-*^ mice were treated with or without 25U/ml of Ifnγ in presence or absence of 250μM of SNAP or different doses of DETA/NO for 36h. The amounts of nitrite (A) and number of cell aggregates per field (C) were quantified. Representative bright field images were acquired at 20X magnification (B). The data is representative of at least three mice per condition. The significance with respect to untreated C57BL/6 control, untreated *Nos2* ^*- /-*^control and Ifnγ treated *Nos2* ^*-/-*^ control are represented as *, τ and θ respectively. **Fig. C in S1 File. Lack of Nos2 does not impact M1-related responses in APECs.** The comparative analysis of cell surface MHC class I (A), intracellular Nos2 (B), Arginase1 (C), CD80 (D), CD86 (E) in APECs, untreated or 25 U/ml of Ifnγ treated for 36 h, from C57BL/6 and *Nos2* ^*-/-*^ mice. The data is represented as mean ± S.E from three independent experiments. Significance is represented as *, Δ and θ when compared to untreated C57BL/6 controls, untreated *Nos2* ^*-/-*^ controls and Ifnγ treated C57BL/6 APECs respectively. **Fig. D in S1 File. Manual tracking of APECs from C57BL/6 and *Nos2*** ^***-/-***^ **mice upon different treatments.** Manual tracking of APECs from C57BL/6 mice treated without or with 25 U/ml of Ifnγ between 18–24 h of Ifnγ addition. APECs from C57BL/6 pretreated for 6 h with Cyt D (10 μM) and Col (1 μg/ml) before manually tracking them between 6–12 h post treatment (A). Manual tracking of APECs from *Nos2* ^*-/-*^ mice treated without or with 25 U/ml of Ifnγ between 18–24 h of Ifnγ addition. Also, *Nos2* ^*-/-*^ APECs pretreated in the presence of 5 μM of SNAP (SNAP^lo^) and 100 μM of SNAP (SNAP^hi^) for 12 h and without or with 25 U/ml of Ifnγ before manually tracking the cells between 18–24 h post Ifnγ addition (B). Represented are results of manual tracking of cells from the same field for above mentioned conditions. The data is representative of two independent experiments. **Fig. E in S1 File. Nitric oxide stabilizes cortical Actin and Tubulin arrangement in APECs treated with Ifnγ.** APECs from C57BL/6 and *Nos2* ^*-/-*^ mice were treated without or with 25 U/ml of Ifnγ in the absence or presence of 100 μM of SNAP for 36 h followed by intracellular staining for F-Actin and α-Tubulin. Confocal images of stained APECs were obtained. The number of cells in each field with cortical Actin and Tubulin arrangement were counted and represented as percentage with respect to total number of cells present in each field. Each point in the graph is the average across multiple fields per condition and three such experiments were analyzed. The data is represented as mean ± S.E from three independent experiments. Significance is represented as *, θ and # when compared to untreated C57BL/6 controls, Ifnγ treated C57BL/6 APECs and Ifnγ treated *Nos2* ^*-/-*^ APECs respectively. **Fig. F in S1 File. Phagocytosis and *S*. Typhimurium uptake in APECs is dependent on Nos2 and Actin polymerization.** The analysis of phagocytosis of latex beads by C57BL/6 APECs, untreated or treated with LNMA (250 μM for 24 h) or Cyt D (10 μM for 6 h) or Col (1 μg/ml for 6 h) (A). The phagocytosis of latex beads by C57BL/6 and *Nos2* ^*-/-*^ APECs represented as percentage phagocytosis with respect to C57BL/6 controls (B). The analysis of intracellular *S*. Typhimurium load in C57BL/6 and *Nos2* ^*-/-*^ APECs 2 h post *S*. Typhimurium infection (C). The data is represented as % phagocytosis in comparison to APECs from C57BL/6 mice in each panel. The analysis of intracellular *S*. Typhimurium load in C57BL/6 APECs 24 h post the indicated MOIs of *S*. Typhimurium infection (D). The data is represented as mean ± S.E from two independent experiments. **Fig. G in S1 File. Nos2 does not affect APECs population upon *S*. Typhimurium infection in mice.** The percentage positive B220 (A), F4/80 (B) and CD11b (C) cells in APECs from C57BL/6 and *Nos2* ^*-/-*^ mice that were uninfected or infected with *S*. Typhimurium for 4 days and analyzed by flow cytometry using specific antibodies to the above mentioned cell surface molecules. The data is represented as mean ± S.E from two independent experiments.
(DOCX)

S1 Video The untreated C57BL/6 APECs captured from 18–24 h post incubation. The images were acquired every 5 min and the movie was made at the rate of 9 frames per second.
(MPG)

S2 Video Ifnγ (25 U/ml) treated C57BL/6 APECs captured from 18–24 h post treatment. The images were acquired every 5 min and the movie was made at the rate of 9 frames per second.
(MPG)

S3 Video The untreated *Nos2* ^*-/-*^ APECs captured from 18–24 h post incubation. The images were acquired every 5 min and the movie was made at the rate of 9 frames per second.
(MPG)

S4 Video Ifnγ (25 U/ml) treated *Nos2* ^*-/-*^ APECs captured from 18–24 h post incubation. The images were acquired every 5 min and the movie was made at the rate of 9 frames per second.
(MPG)

S5 Video Cytochalsin D (10 μM) treated C57BL/6 APECs captured from 6–12 h post treatment. The images were acquired every 5 min and the movie was made at the rate of 9 frames per second.
(MPG)

S6 Video Colcemid (1 μg/ml) treated C57BL/6 APECs captured from 6–12 h post treatment. The images were acquired every 5 min and the movie was made at the rate of 9 frames per second.
(MPG)
