# Supplementary figures and images for: Interferon-Gamma and Nitric Oxide Synthase 2 Mediate the Aggregation of Resident Adherent Peritoneal Exudate Cells: Implications for the Host Response to Pathogens
Source: PLoS One. 2015 Jun 1;10(6):e0128301. doi: 10.1371/journal.pone.0128301 (PMC4452304; doi:10.1371/journal.pone.0128301)

**Supplementary figures**


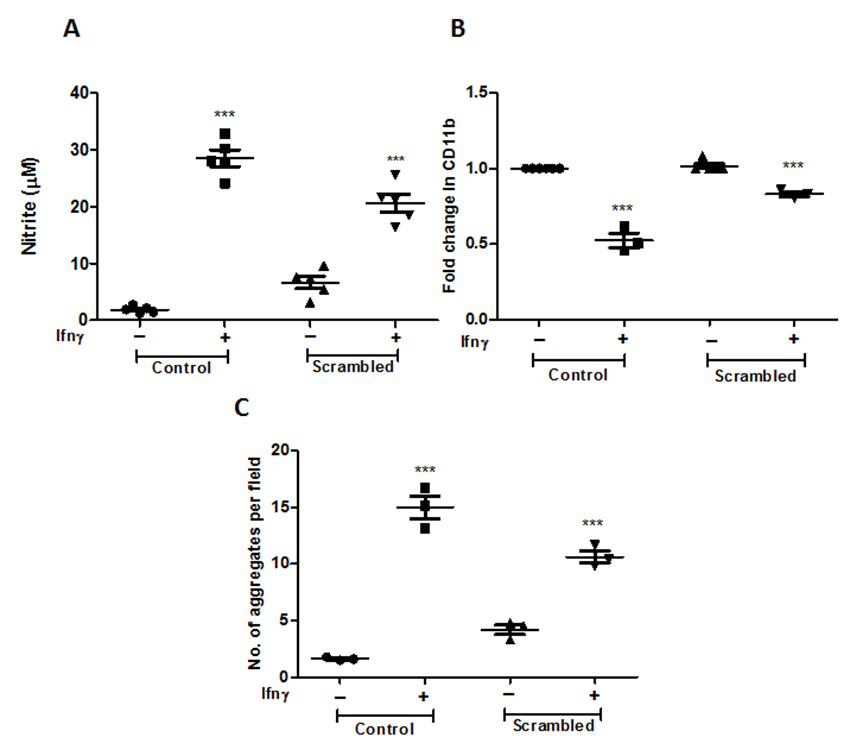


**Fig. A**


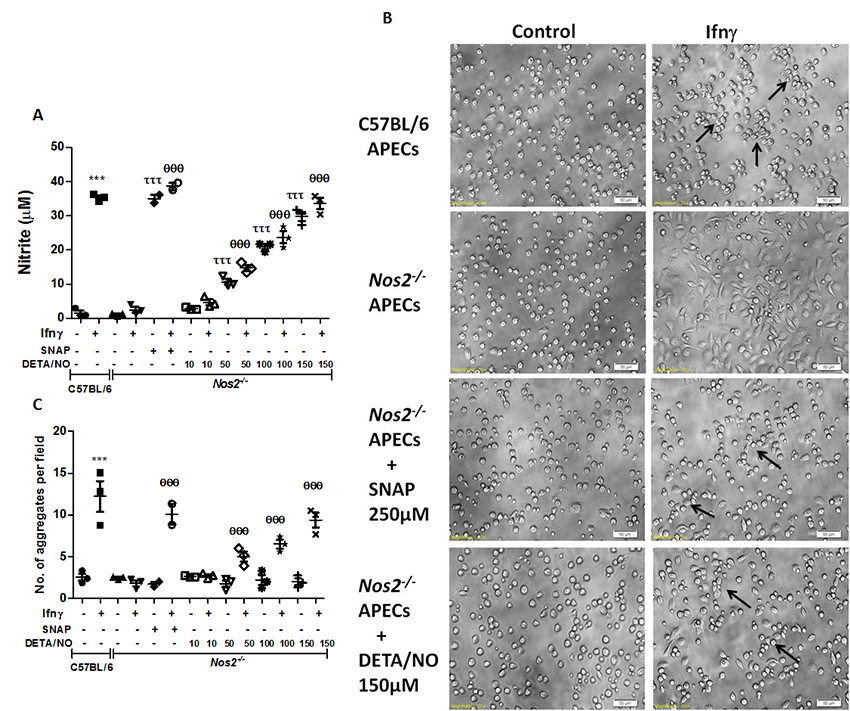


**Fig. B**


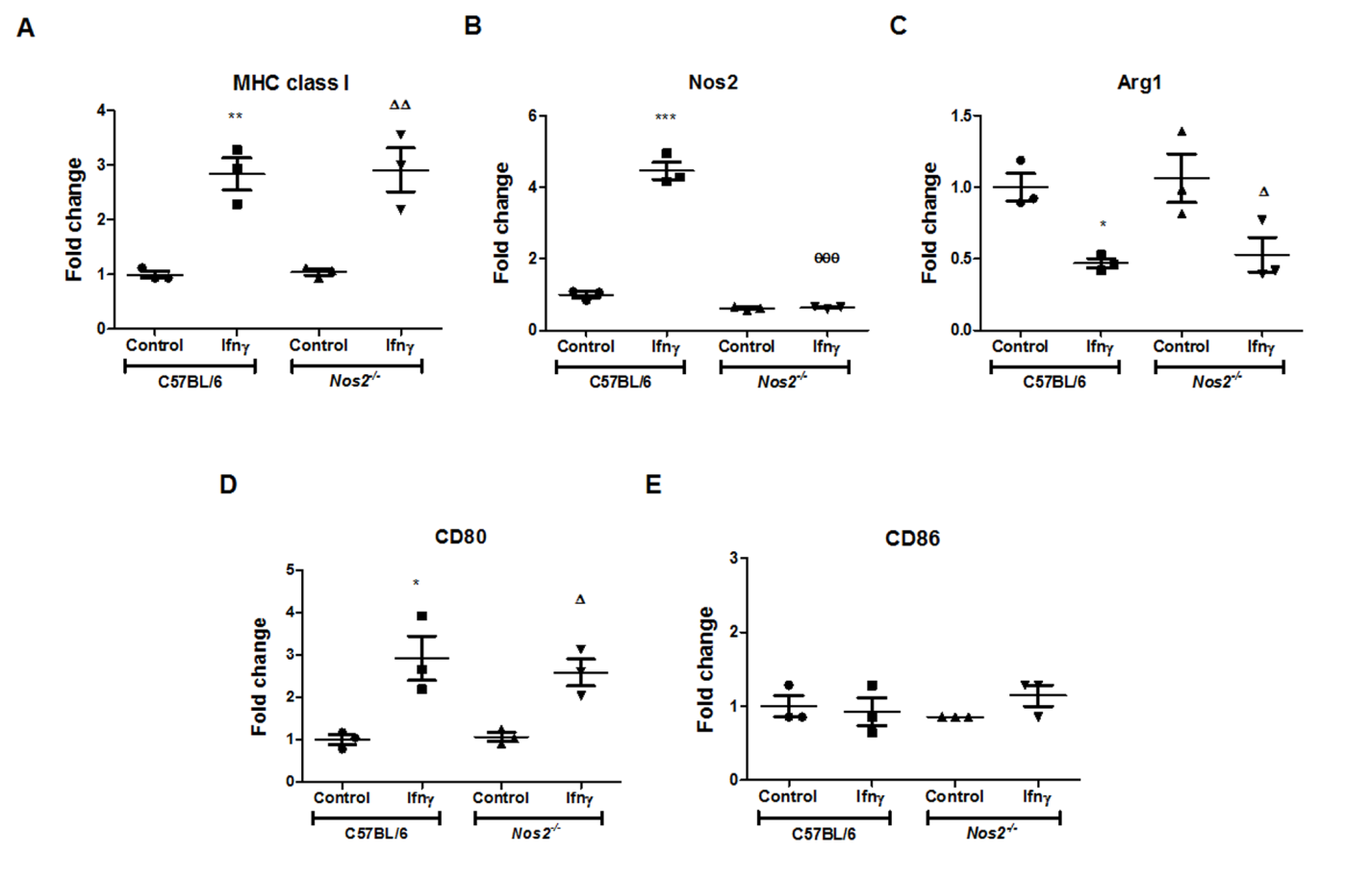


**Fig. C**


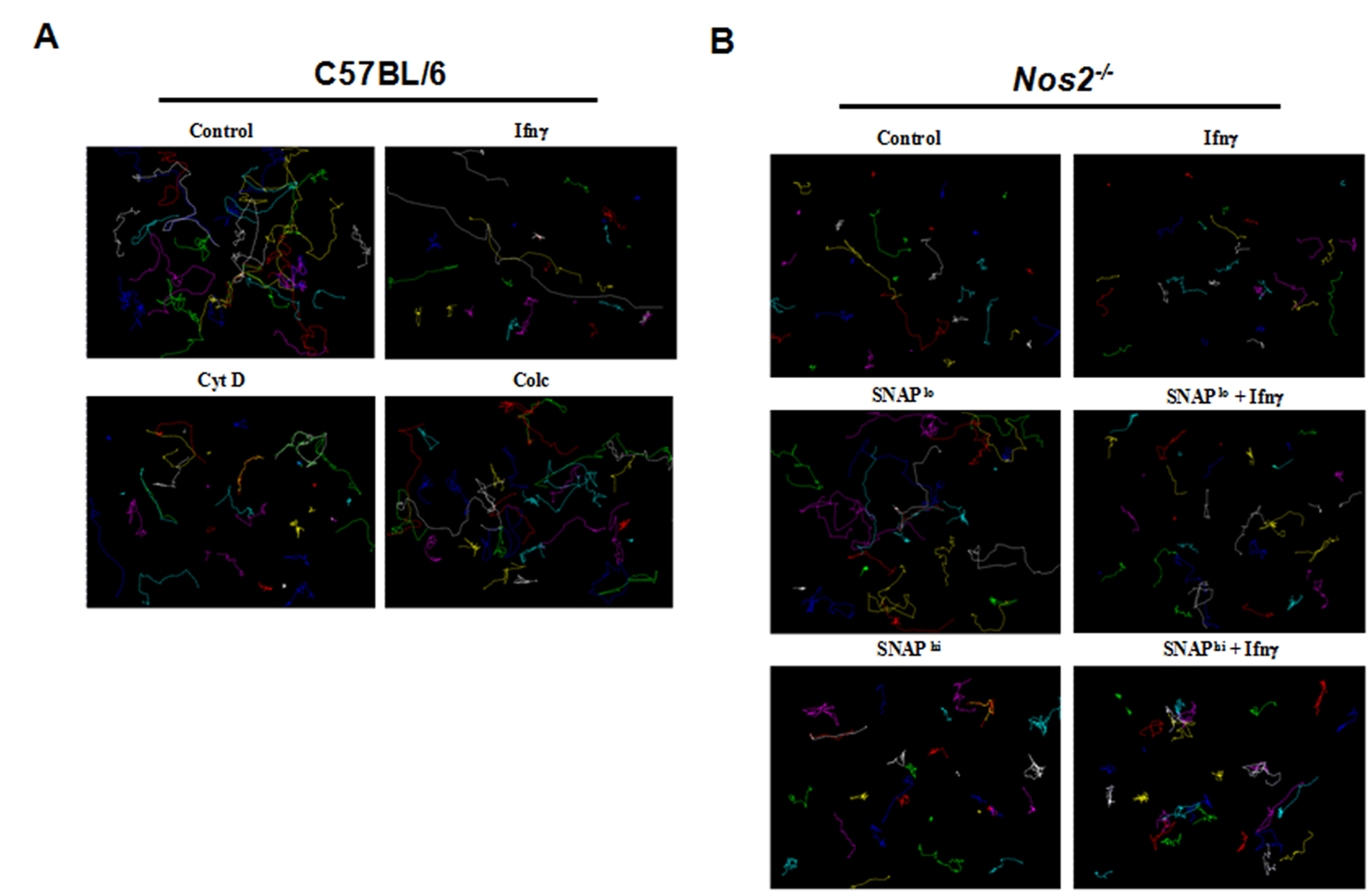


**Fig. D**


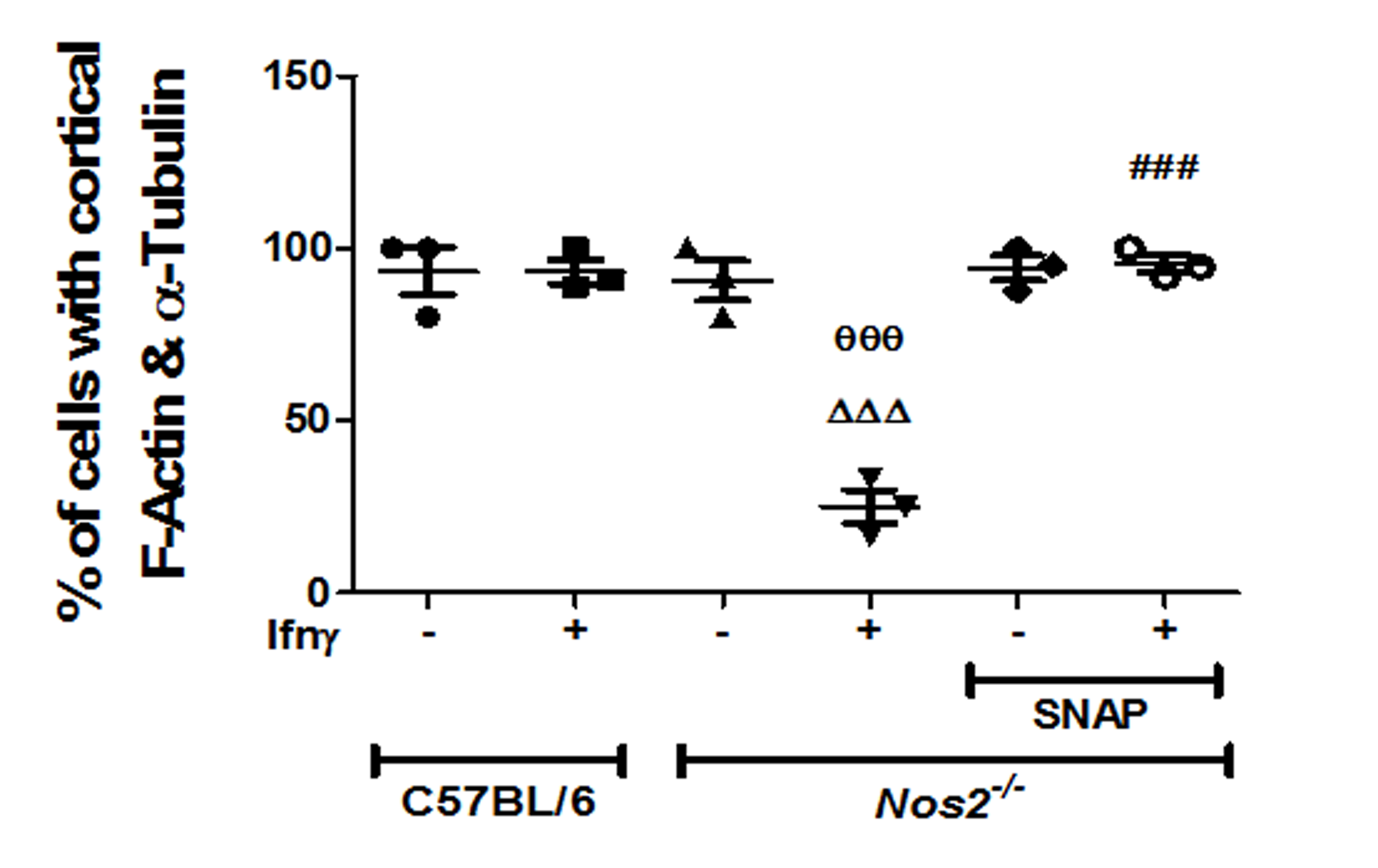


**Fig. E**


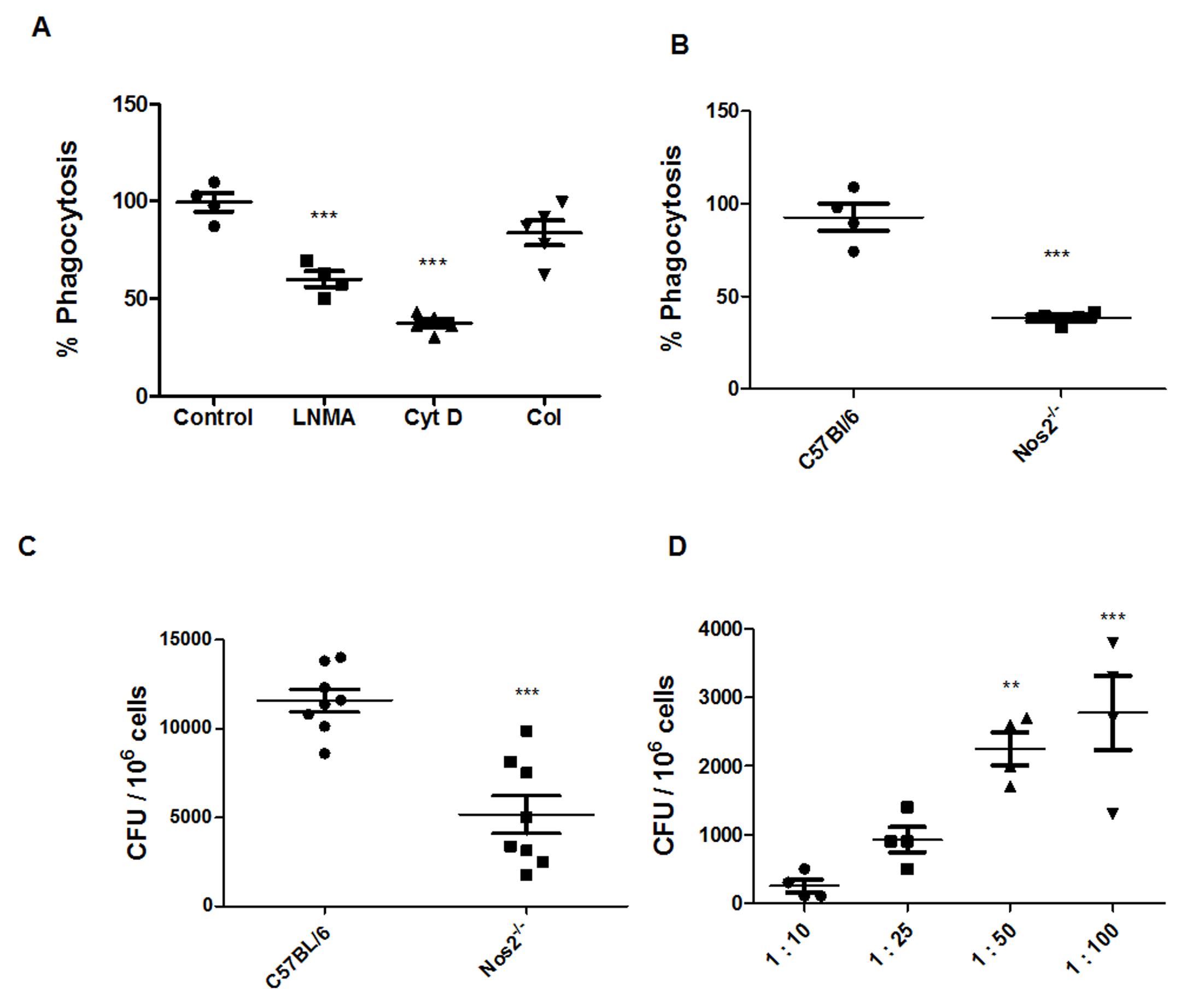


**Fig. F**


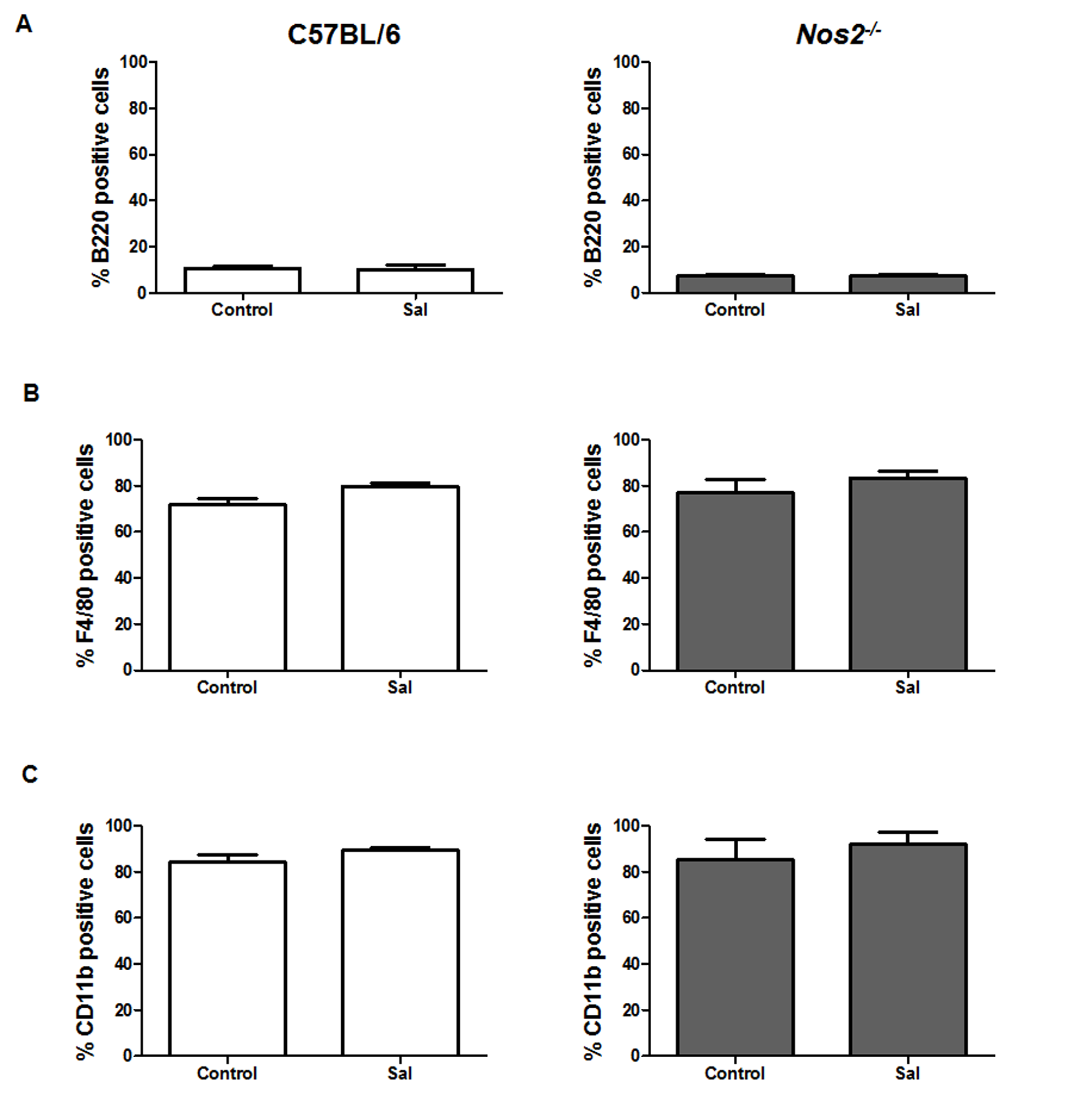


**Fig. G**

Supplement: S1 File — Fig. A in S1 File. Transfection with scrambled siRNA does not alter major responses of APECs. APECs from C57BL/6 with or without transfection with 250nM scrambled siRNA were treated in the presence or absence of 25U/ml of Ifnγ for 24h. The amounts of nitrite (A), Fold change in cell surface CD11b expression (B) and number of cell aggregates per field (C) were quantified. The data is representative of at least three different mice in each condition. The significance with respect to untransfected control are represented as *. Fig. B in S1 File. DETA/NO induces aggregation in Nos2 -/- APECs upon Ifnγ treatment. APECs from C57BL/6 and Nos2 -/- mice were treated with or without 25U/ml of Ifnγ in presence or absence of 250μM of SNAP or different doses of DETA/NO for 36h. The amounts of nitrite (A) and number of cell aggregates per field (C) were quantified. Representative bright field images were acquired at 20X magnification (B). The data is representative of at least three mice per condition. The significance with respect to untreated C57BL/6 control, untreated Nos2 - /-control and Ifnγ treated Nos2 -/- control are represented as *, τ and θ respectively. Fig. C in S1 File. Lack of Nos2 does not impact M1-related responses in APECs. The comparative analysis of cell surface MHC class I (A), intracellular Nos2 (B), Arginase1 (C), CD80 (D), CD86 (E) in APECs, untreated or 25 U/ml of Ifnγ treated for 36 h, from C57BL/6 and Nos2 -/- mice. The data is represented as mean ± S.E from three independent experiments. Significance is represented as *, Δ and θ when compared to untreated C57BL/6 controls, untreated Nos2 -/- controls and Ifnγ treated C57BL/6 APECs respectively. Fig. D in S1 File. Manual tracking of APECs from C57BL/6 and Nos2 -/- mice upon different treatments. Manual tracking of APECs from C57BL/6 mice treated without or with 25 U/ml of Ifnγ between 18–24 h of Ifnγ addition. APECs from C57BL/6 pretreated for 6 h with Cyt D (10 μM) and Col (1 μg/ml) before manually tra [file pone.0128301.s001.docx]
